# BRF1 accelerates prostate tumourigenesis and perturbs immune infiltration

**DOI:** 10.1038/s41388-019-1106-x

**Published:** 2019-11-18

**Authors:** Carolyn J. Loveridge, Sarah Slater, Kirsteen J. Campbell, Noor A. Nam, John Knight, Imran Ahmad, Ann Hedley, Sergio Lilla, Peter Repiscak, Rachana Patel, Mark Salji, Janis Fleming, Louise Mitchell, Colin Nixon, Douglas Strathdee, Matthew Neilson, Chara Ntala, Sheila Bryson, Sara Zanivan, Joanne Edwards, Craig N. Robson, Carl S. Goodyear, Karen Blyth, Hing Y. Leung

**Affiliations:** 1 Institute of Cancer Sciences, College of Medical, Veterinary and Life Sciences, University of Glasgow, Bearsden, Glasgow, G61 1QH UK; 2 CRUK Beatson Institute, Bearsden, Glasgow, G61 1BD UK; 3 Northern Institute for Cancer Research, The Medical School, Newcastle University, Framlington Place, Newcastle upon Tyne, NE2 4HH UK; 4 Institute of Infection, Immunity and Inflammation, College of Medical, Veterinary and Life Sciences, University of Glasgow, Glasgow, G12 8TA UK; 5 Present Address: Department of Basic Sciences and Oral Biology, Faculty of Dentistry, Universiti Sains Islam Malaysia, Kuala Lumpur, Malaysia

**Keywords:** Urological cancer, Cancer

## Abstract

BRF1 is a rate-limiting factor for RNA Polymerase III-mediated transcription and is elevated in numerous cancers. Here, we report that elevated levels of BRF1 associate with poor prognosis in human prostate cancer. In vitro studies in human prostate cancer cell lines demonstrated that transient overexpression of BRF1 increased cell proliferation whereas the transient downregulation of BRF1 reduced proliferation and mediated cell cycle arrest. Consistent with our clinical observations, *BRF1* overexpression in a *Pten*-deficient mouse (*Pten*^*Δ/Δ*^ *BRF1*^*Tg*^) prostate cancer model accelerated prostate carcinogenesis and shortened survival. In *Pten*^*Δ/Δ*^ *BRF1*^*Tg*^ tumours, immune and inflammatory processes were altered, with reduced tumoral infiltration of neutrophils and CD4 positive T cells, which can be explained by decreased levels of complement factor D (CFD) and C7 components of the complement cascade, an innate immune pathway that influences the adaptive immune response. We tested if the secretome was involved in BRF1-driven tumorigenesis. Unbiased proteomic analysis on BRF1-overexpresing PC3 cells confirmed reduced levels of CFD in the secretome, implicating the complement system in prostate carcinogenesis. We further identify that expression of *C7* significantly correlates with expression of *CD4* and has the potential to alter clinical outcome in human prostate cancer, where low levels of *C7* associate with poorer prognosis.

## Introduction

The BRF1 transcription factor functions together with TATA-binding protein and B double-prime 1 in a complex called TFIIIB. BRF1 is required to recruit RNA polymerase III (Pol III) to target genes and is rate-limiting for Pol III-mediated transcription [1]. Increased protein synthesis plays a key role in oncogenesis and Pol III products, including transfer RNAs (tRNAs) and other short non-coding RNAs, such as the ribosomal component 5S RNA, are essential for protein synthesis. Major components of the ribosome (ribosomal proteins e.g. RPS-19, -21, -24 and ribosomal RNA) are upregulated in prostate cancer (PCa) [2, 3]. BRF1 expression is elevated and associated with poor prognosis in hepatocellular, breast and gastric cancers [4–6] but its role in PCa remains unclear.

PCa is the third most common cause of cancer-associated death in men worldwide and progression is unpredictable [7]. Patients with similar tumour grade and histology can quickly progress to incurable, advanced metastatic disease or alternatively they can survive for decades with local, indolent, disease [8]. Evasion of surveillance and recognition by the innate and adaptive immune system are implicated in PCa progression [9] and it has been proposed that oncogenic drivers can contribute to immunoresistance in PCa [10]. There is an unmet need to better understand these biological processes in PCa which tends to resist available immune checkpoint inhibitors.

The complement pathway links innate and adaptive immunity to coordinate appropriate immune responses [11]. Complement activation occurs via the classical, lectin or alternative pathways [12], culminating in the formation of the membrane attack complex (MAC) (composed of C5b-C9) which punctures cell membranes to cause cytolysis. The concentration of C7 at sites of complement activation is a limiting factor for MAC formation [13]. Complement activation recruits neutrophils, monocytes and macrophages to sites of inflammation [14], promotes opsonisation of B-cell responses [12] and enhances T-cell responses (activation, differentiation, polarisation and apoptosis) [11, 12].

Here, we show that elevated BRF1 associates with poor prognosis in PCa and accelerates prostate tumorigenesis in a new genetically engineered mouse model. For the first time, we demonstrate that elevated BRF1 expression in the prostatic epithelium can impact upon the tumour microenvironment in vivo.

## Results

### BRF1 is elevated in prostate cancer and confers a poor prognosis

**Fig. 1:**
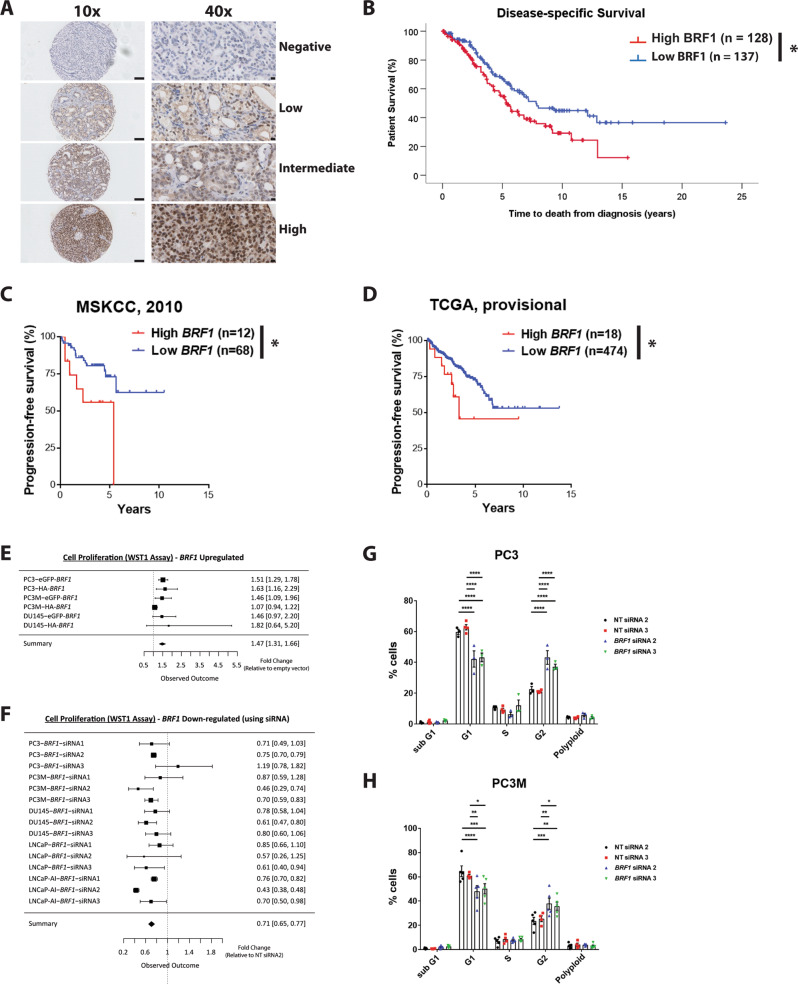
BRF1 is a prognostic marker in prostate cancer and mediates effects on cellular proliferation and the cell cycle in vitro. **a** Example images of PCa cores stained for BRF1 with varying Histoscores: negative (score 0), low (score 125), intermediate (score 175) or high (score 300). Higher magnification images show finer detail of staining from the same cores. Scale bars are shown (100 µm for lower magnification images; 10 µm for higher magnification images). **b** Kaplan–Meier plot for disease-specific survival of PCa patients stratified according to low (below median histoscore; *n* = 128) versus high (above median histoscore; *n* = 137) expression of BRF1 within PCa cohort. **c** Kaplan–Meier plot for progression-free survival of patients in MSKCC (2010) dataset segregated for low and high *BRF1* expression as indicated in oncoprint in Fig. S1c. **d** Kaplan–Meier plot for progression-free survival of patients in TCGA (provisional) dataset segregated for low and high *BRF1* expression as indicated in oncoprint in Fig. S1d. Log-rank (Mantel–Cox) Test was performed to compare survival curves; *p < 0.05. **e** Forest plot summarising WST1 cell proliferation assay results from PC3, PC3M and DU145 (all *n* = 3) cells which were transiently transfected with HA-BRF1, GFP-BRF1 and their respective HA- and GFP-empty vector controls for 48 h. **f** Forest plot summarising WST1 cell proliferation assay results from PC3 (*n* = 4), PC3M, DU145, LNCaP and LNCaP AI (all *n* = 3) cells which were transiently transfected with three independent siRNAs for BRF1 and control non-targeting (NT) siRNA for 48 h. In presented Forest plots in **e** and **f**, each box represents the sample mean [relative to empty vector (**e**) or control NT siRNA (**f**)]; error bars represent 95% confidence intervals; the centre of the diamond in summary line represents the collective sample mean; the width of the diamond represents the 95% confidence interval for the collective sample mean. PC3 (**g**) and PC3M (**h**) cells were transiently transfected with two independent siRNAs for *BRF1* and two different control NT siRNAs for 72 h then subjected to bromodeoxyuridine (BrdU)- and propidium iodide (PI)-labelling followed by flow cytometry analysis to determine cell cycle positions. Data presented are from *n* = 3 (PC3 cells: NT siRNA 2; *BRF1* siRNA’s 2 and 3), *n* = 4 (PC3 cells: NT siRNA 3; PC3M cells: NT siRNA 3) or *n* = 5 (PC3M cells: NT siRNA 2; *BRF1* siRNA’s 2 and 3) experiments. Individual data points are shown in the presented graphs; long horizontal lines indicate the Mean; error bars represent SEM; 2way ANOVA was used to calculate *p* values; *p < 0.05; **p < 0.01; ***p < 0.001; ****p < 0.0001 from NT siRNA 2 or 3

BRF1 immunoreactivity was studied in 516 PCa cases and 134 benign prostatic hyperplasia (BPH) controls. Nuclear BRF1 immunoreactivity (Fig. 1a) was significantly upregulated in PCa (*p* = 0.0032) (Fig. S1a, Table S1) and in clinically significant disease (Gleason sum score = 7 and > 7: *p* = 0.0039 and 0.0091, respectively) (Fig. S1b, Table S2) when compared with BPH. Elevated BRF1 expression correlated with shorter disease-specific survival (5.39 vs. 7.81 years; *p* = 0.012) (Fig. 1b, Table S3). Utilising the Memorial Sloan-Kettering Cancer Center (MSKCC) (2010) [15] and The Cancer Genome Atlas (TCGA) (provisional) prostate adenocarcinoma datasets in cBioPortal [16, 17], elevated *BRF1* expression also associated with poor outcome (Figs. 1c, d and S1c, d; Tables S4 and S5).

### BRF1 drives in vitro cellular proliferation and cell cycle progression

BRF1 expression was assessed in human PCa cell lines (Fig. S1e). Ectopic overexpression of *BRF1*, using HA-*BRF1* or eGFP-*BRF1* plasmids, mediated a modest but significant increase in cell proliferation in PC3, PC3M and DU145 cells by WST1 assay (Figs. 1e, S2a and S3a; Table S6). Conversely, downregulation of *BRF1* expression in PCa cells (with at least two out of three independent siRNAs) reduced proliferation (Figs. 1f, S2b and S3b; Table S7), with FACS analysis revealing reduced G1 subpopulation and increased G2 phase (Fig. 1g, h). Collectively, manipulation of BRF1 expression alters proliferation in vitro.

### Increased *BRF1* accelerates prostate tumourigenesis in vivo

**Fig. 2:**
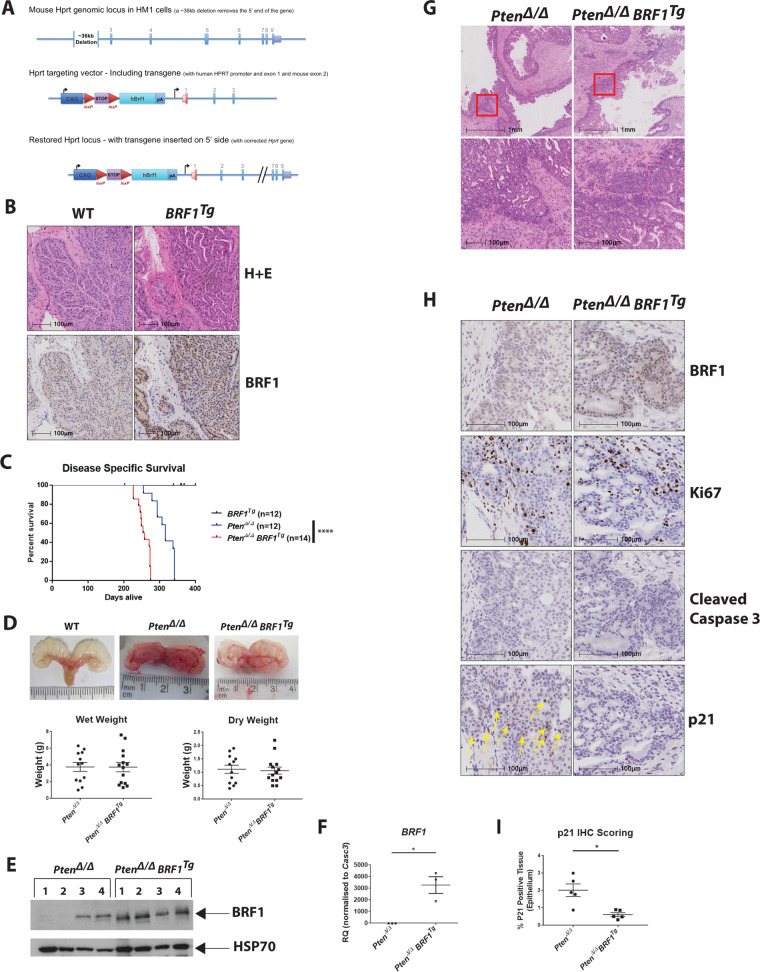
Double mutant *Pten*^*Δ/Δ*^ *BRF1*^*Tg*^ mice have reduced survival compared with *Pten*^*Δ/Δ*^ mice. **a** Illustration of strategy for targeting overexpression of human *BRF1* gene. The mouse *Hprt* genomic locus, the *Hprt* targeting vector (including the human *BRF1* transgene) and the restored *Hprt* locus (with transgene inserted on 5’ side) are shown. **b** Representative micrographs of H + E and BRF1 IHC staining in anterior prostate tissue from WT and *BRF1*^*Tg*^ mice (*n* = 5 for each genotype). Scale bars are shown (100 µm for all images). **c** Kaplan–Meier plot representing disease-specific survival of *BRF1*^*Tg*^ (*n* = 12), *Pten*^*Δ/Δ*^ (*n* = 12) and *Pten*^*Δ/Δ*^ *BRF1*^*Tg*^ (*n* = 14) mice. Log-rank (Mantel–Cox) Test was performed to compare *Pten*^*Δ/Δ*^ and *Pten*^*Δ/Δ*^ *BRF1*^*Tg*^ survival curves; ****p < 0.0001. **d** Representative images of isolated prostates from *Pten*^*Δ/Δ*^ and *Pten*^*Δ/Δ*^ *BRF1*^*Tg*^ mice that had reached clinical endpoint and a wild type (WT) mouse taken at an equivalent age (top panel). Isolated prostates from *Pten*^*Δ/Δ*^ (*n* = 12) and *Pten*^*Δ/Δ*^ *BRF1*^*Tg*^ (*n* = 14) mice were weighed prior to removal of cystic fluid (termed Wet Weight), then were re-weighed to assess the solid tumour mass (termed Dry Weight) (bottom panel). **e** Whole-cell lysates prepared from prostate tumour tissue obtained from *Pten*^*Δ/Δ*^ (*n* = 4) and *Pten*^*Δ/Δ*^ *BRF1*^*Tg*^ (*n* = 4) mice were subjected to SDS-PAGE, followed by western blotting using an anti-BRF1 antibody. HSP70 served as a loading control. **f** qPCR analysis of RNA isolated from prostate tumour tissue obtained from *Pten*^*Δ/Δ*^ (*n* = 3) and *Pten*^*Δ/Δ*^ *BRF1*^*Tg*^ (*n* = 3) mice specifically for the human *BRF1* transgene. *Casc3* was used as a reference gene for normalisation. **g** Representative micrographs of H + E staining in anterior prostate tissue from *Pten*^*Δ/Δ*^ and *Pten*^*Δ/Δ*^ *BRF1*^*Tg*^ mice (*n* = 5 for each genotype). Red box in lower magnification images in upper panel highlights region shown in higher magnification images in lower panel. Scale bars are shown (1 mm for upper panel images; 100 µm for bottom panel images). **h** Representative micrographs of BRF1, Ki67, Cleaved Caspase-3 and p21 IHC staining in anterior prostate tumour tissue from *Pten*^*Δ/Δ*^ and *Pten*^*Δ/Δ*^ *BRF1*^*Tg*^ mice (*n* = 5 for each genotype). Yellow arrows highlight p21 staining. Scale bars are shown (100 µm for all images). **i** Scoring of p21 IHC staining. p21 IHC staining was analysed in 25 manually annotated areas of prostate tumour epithelium per slide from *Pten*^*Δ/Δ*^ (*n* = 5) and *Pten*^*Δ/Δ*^ *BRF1*^*Tg*^ (*n* = 5) mice using HALO software (see methods). **d,** **f,** **i** Individual data points are shown in the presented graphs; long horizontal line indicates the Mean; error bars represent SEM; Welch’s *t* test (unpaired, 2 tailed) was used to calculate *p* value; *p < 0.05

An in vivo model with prostate epithelium-specific overexpression of BRF1 was generated by inter-crossing *BRF1* mice [18] with the *Pbsn* (PB)-Cre4 strain [19] to generate PB*-*Cre4:*BRF1* (herein referred to as *BRF1*^*Tg*^) mice (see Supplementary methods; Fig. 2a). Male *BRF1*^*Tg*^ mice were aged to over 1 year, and no malignancy or alteration in prostate architecture was observed (*n* = 12 WT; *n* = 12 *BRF1*^*Tg*^) (Fig. 2b). We then crossed *BRF1*^*Tg*^ with *Pten*^*fl/fl*^ [20] mice to generate prostate epithelium-specific double mutant PB*-*Cre4:*Pten*^*fl/fl*^ *BRF1* (herein referred to as *Pten*^*Δ/Δ*^ *BRF1*^*Tg*^) mice. Survival of PB*-*Cre4:*Pten*^*fl/fl*^ (herein referred to as *Pten*^*Δ/Δ*^) control mice was consistent with our previous report [21]. *Pten*^*Δ/Δ*^ *BRF1*^*Tg*^ mice had significantly shorter disease-specific survival compared with *Pten*^*Δ/Δ*^ siblings (median 256 vs 316 days, respectively; *p* < 0.0001) (Fig. 2c, Table S8), with comparable endpoint tumour weights (Fig. 2d, Table S9) despite being harvested earlier. Interestingly, whilst high BRF1 protein levels were observed in all *Pten*^*Δ/Δ*^ *BRF1*^*Tg*^ tumours, some tumours from *Pten*^*Δ/Δ*^ mice also had elevated BRF1 expression (Fig. 2e). Quantitative real-time PCR (qPCR) was performed using primers specific for human *BRF1* confirmed its overexpression in *Pten*^*Δ/Δ*^ *BRF1*^*Tg*^ prostate tumours (*p* = 0.0459) (Fig. 2f). *Pten*^*Δ/Δ*^ and *Pten*^*Δ/Δ*^ *BRF1*^*Tg*^ tumours were found to be histologically similar (Fig. 2g). High BRF1 expression was found in the prostate epithelium of *BRF1*^*Tg*^ (Fig. 2b) and *Pten*^*Δ/Δ*^ *BRF1*^*Tg*^ (Fig. 2h) mice as expected. Although levels of Ki67 and cleaved caspase-3, markers for proliferation and apoptosis, respectively, were similar (Fig. S4a, b), we observed a significant reduction in the expression of the cell cycle inhibitor p21 in *Pten*^*Δ/Δ*^ *BRF1*^*Tg*^ tumours (*p* = 0.0151) (Fig. 2h, i), consistent with the cell cycle data in Fig. 1g, h. Together these data suggest that increased expression of *BRF1* alone does not drive PCa but can co-operate with *Pten* loss.

### *Pten*^*Δ/Δ*^*BRF1*^*Tg*^ prostate tumours harbour alterations in immune system processes

**Fig. 3:**
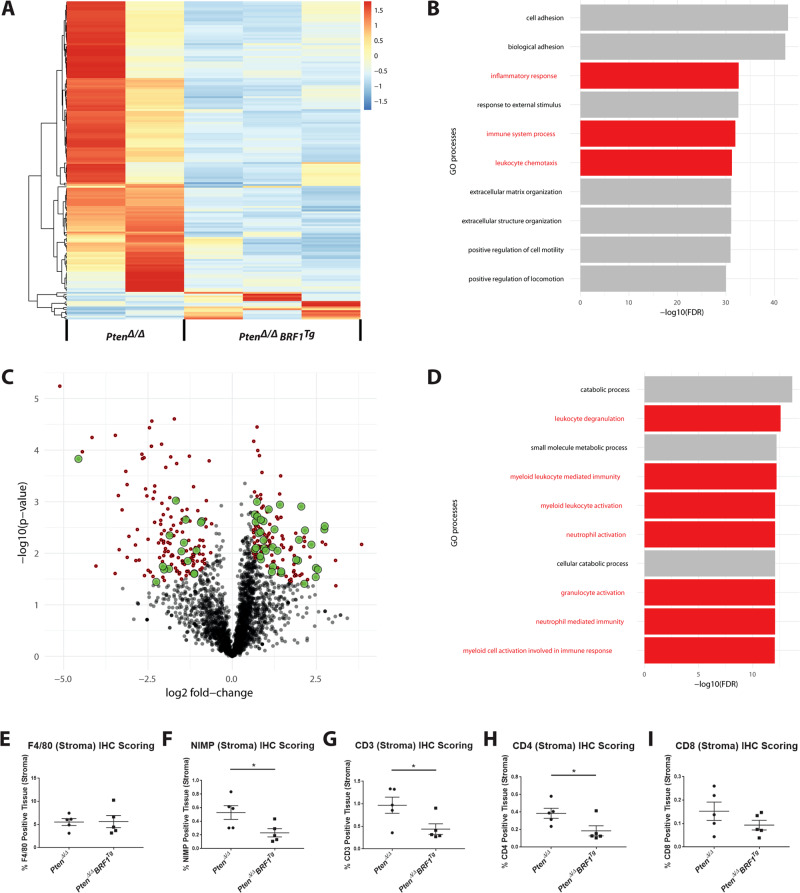
Transcriptomics and proteomics reveal that immune system processes are significantly altered in *Pten*^*Δ/Δ*^ *BRF1*^*Tg*^ mice and infiltration of neutrophils and CD4 positive T lymphocytes is significantly reduced in *Pten*^*Δ/Δ*^ *BRF1*^*Tg*^ compared with *Pten*^*Δ/Δ*^ mice. **a** Heatmap of RNA-Seq data. Presented are the 174 genes that are implicated in immune processes which have significantly altered expression (>1.5-fold change; *p*.adj < 0.05) between *Pten*^*Δ/Δ*^ and *Pten*^*Δ/Δ*^ *BRF1*^*Tg*^ prostate tumours. Because of a technical problem, the library preparation step for one *Pten*^*Δ/Δ*^ sample failed resulting in *n* = 2 for *Pten*^*Δ/Δ*^ and *n* = 3 for *Pten*^*Δ/Δ*^ *BRF1*^*Tg*^. In the heatmap, blue represents downregulation of gene expression (Row Z-Score < 0); red represents up-regulation of gene expression (Row Z-Score > 0). **b** Bar chart illustrating results of gene enrichment analysis from GeneGO MetaCore of RNA-Seq data – the top 10 significantly altered GO processes are shown; highlighted in red are those which relate to the immune response. **c** Volcano plot of proteomic data from comparison of *Pten*^*Δ/Δ*^ (*n* = 4) and *Pten*^*Δ/Δ*^ *BRF1*^*Tg*^ (*n* = 3) prostate tumour samples. Highlighted in red are proteins whose abundance was found to be significantly (fold change > 1.5; *p*.adj < 0.05) altered between *Pten*^*Δ/Δ*^ and *Pten*^*Δ/Δ*^ *BRF1*^*Tg*^ samples; amongst these significantly altered proteins, highlighted in green are those that related to the immune process GO term (Table S10). **d** Bar chart illustrating results of gene enrichment analysis from GeneGO MetaCore of proteomic data – the top 10 significantly altered GO processes are shown; highlighted in red are those which relate to the immune response. Analysis of IHC staining for F4/80 (**e**), NIMP (**f**), CD3 (**g**), CD4 (**h**) and CD8 (**i**). Total observable prostate stromal tissue per slide was manually annotated on each sample from *Pten*^*Δ/Δ*^ (*n* = 5) and *Pten*^*Δ/Δ*^ *BRF1*^*Tg*^ (*n* = 5) mice for each marker then staining within these annotated areas was analysed using HALO software (see supplementary information). **e**–**i** Individual data points are shown in the presented graphs; long horizontal line indicates the Mean; error bars represent SEM; Welch’s *t* test (unpaired, 2 tailed) was used to calculate *p* value; *p < 0.05

RNA-Sequencing (RNA-Seq) identified 522 significantly altered genes (fold change > 1.5; *p*.adj < 0.05) between *Pten*^*Δ/Δ*^ and *Pten*^*Δ/Δ*^ *BRF1*^*Tg*^ endpoint tumours, with 174 coding genes involved in immune processes (see heatmap in Fig. 3a; Table S10)—these are predominantly downregulated in *Pten*^*Δ/Δ*^ *BRF1*^*Tg*^ tumours. Gene enrichment analysis further identified immune system processes, inflammatory response and leukocyte chemotaxis amongst the most overrepresented biological processes (Fig. 3b and Table S11).

Untargeted proteomics analysis on the *Pten*^*Δ/Δ*^ and *Pten*^*Δ/Δ*^ *BRF1*^*Tg*^ tumours revealed that the abundance of 300 proteins was significantly (fold change > 1.5; *p*.adj < 0.05) altered, with 52 of these being related to immune processes (Fig. 3c, Table S12). Pathway enrichment analysis subsequently revealed that 7 of the top 10 most overrepresented biological processes related to immune responses (Fig. 3d and Table S13).

Finally, analysis of the top 10 overrepresented pathways (Table S14) in both RNA-Seq and proteomics again pointed towards altered immune status as a key difference between *Pten*^*Δ/Δ*^ and *Pten*^*Δ/Δ*^ *BRF1*^*Tg*^ prostate tumours, highlighting aspects of both the innate immune response and the adaptive immune response.

### Infiltration of neutrophils, CD3 and CD4 positive T lymphocytes is significantly reduced in *Pten*^*Δ/Δ*^*BRF1*^*Tg*^ (compared with *Pten*^*Δ/Δ*^) tumours

Analysis of innate immune cells showed no significant difference in the level of macrophages but neutrophils were significantly reduced (*p* = 0.0421) in the stroma (defined in Fig. S5a) of *Pten*^*Δ/Δ*^ *BRF1*^*Tg*^ versus *Pten*^*Δ/Δ*^ prostate tumours (Figs. 3e, f and S5c). Consistent with our previous report [21] and clinical PCa [22], stromal tertiary lymphoid aggregates were present in murine tumours (Fig. S5b), neither the total area of these structures nor markers of B-cell response were unaltered (Figs. S6 and S7a, b). *Pten*^*Δ/Δ*^ *BRF1*^*Tg*^ tumours contained significantly fewer stromal CD3 and CD4 T cells (*p* = 0.0438, 0.0394, respectively) but stromal CD8 or FOXP3 (a marker for regulatory T cells) T cells were not altered (Figs. 3g–i, S5d, c).

Collectively, we identified reduced infiltration of neutrophils and CD4 positive T lymphocytes in *Pten*^*Δ/Δ*^ *BRF1*^*Tg*^ prostate tumours, in keeping with reduced levels of immune/inflammatory response genes (Fig. 3a) and expression of *Cd4* (RNA-Seq data: log2FoldChange = −1.8203; *p*.adj = 0.0067; Table S8) in prostate tumours from *Pten*^*Δ/Δ*^ *BRF1*^*Tg*^ mice.

### *Pten*^*Δ/Δ*^*BRF1*^*Tg*^ tumours have reduced complement pathway activation

**Fig. 4:**
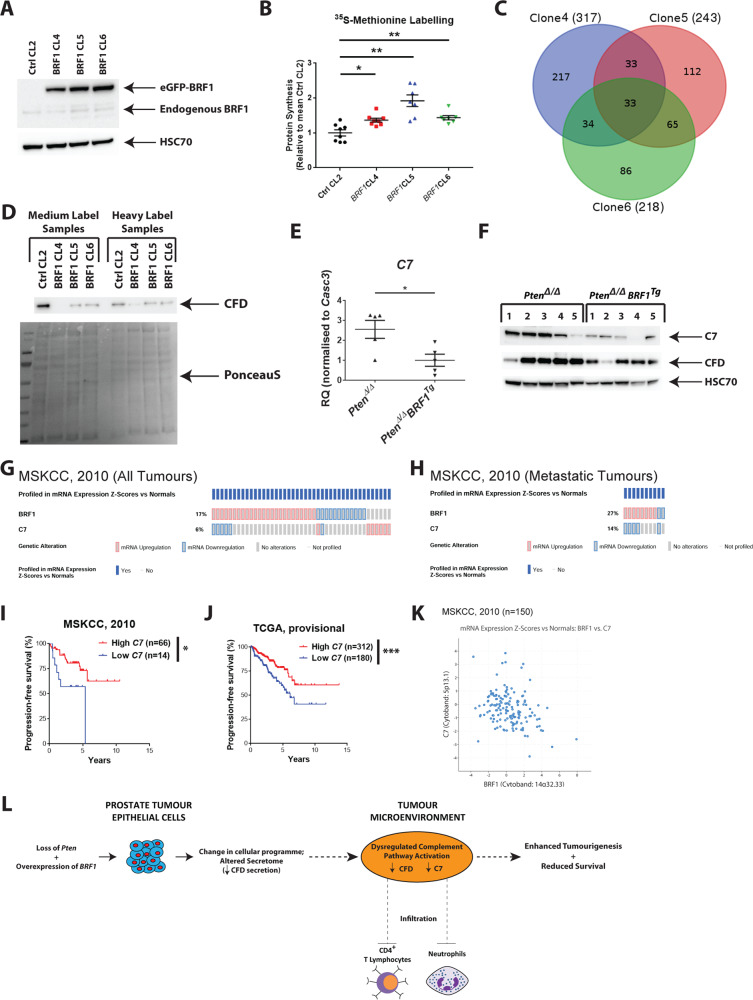
Complement pathway activation is (i) reduced when *BRF1* is overexpressed in vitro, (ii) reduced in prostate tumours from *Pten*^*Δ/Δ*^ *BRF1*^*Tg*^ mice and (iii) inversely correlates with *BRF1* expression in human PCa. **a** Western blotting of whole-cell lysates prepared from PC3 Ctrl CL2 and BRF1 CL4, 5 and 6 cells using anti-BRF1 antibody and HSC70 as a loading control. Blot shown is representative of three independent experiments. **b** Comparison of incorporation of ^35^S-methionine label, which was measured by scintillation counting, between PC3 stable clones (*n* = 3; 2-3 wells per clone were used per each individual experiment). Data from BRF1 CL4, 5 and 6 cells were normalised to the mean of PC3 Ctrl CL2 cells. Individual data points are shown in the presented graph; long horizontal line indicates the Mean; error bars represent SEM; Welch’s *t* test (unpaired, two tailed) with Bonferroni correction for multiple testing was used to calculate *p*.adj values; *p.adj < 0.05; **p.adj < 0.01. **c** Venn diagram outlining numbers of proteins that had >1.2-fold change in abundance in the same direction in PC3 BRF1 overexpressing clones CL4, 5 and 6 compared with Ctrl CL2. **d** Western blotting of purified secreted proteins prepared from PC3 Ctrl. CL2 and PC3 BRF1 overexpressing CL4, 5 and 6 using an anti-CFD antibody. PonceauS staining was used as a loading control. **e** qPCR analysis of isolated RNA prepared from prostate tumour tissue obtained from *Pten*^*Δ/Δ*^ (*n* = 5) and *Pten*^*Δ/Δ*^ *BRF1*^*Tg*^ (*n* = 5) mice for *C7*. *Casc3* was used as a reference gene for normalisation. Individual data points are shown in the presented graph; long horizontal line indicates the Mean; error bars represent SEM; Welch’s *t* test (unpaired, 2 tailed) was used to calculate *p* value; *p < 0.05. **f** Western blotting of whole-cell lysates prepared from prostate tumour tissue obtained from *Pten*^*Δ/Δ*^ (*n* = 5) and *Pten*^*Δ/Δ*^ *BRF1*^*Tg*^ (*n* = 5) mice using anti-C7 and anti-CFD antibodies. HSC70 served as a loading control. Oncoprint from cBioPortal illustrating mRNA expression profile in *BRF1* and *C7* in all tumours (216 cases) (**g**) and metastatic tumours (37 cases) (**h**) in MSKCC (2010) prostate adenocarcinoma dataset. Kaplan–Meier plots for progression-free survival in MSKCC (2010) (**i**) and TCGA (provisional) (**j**) prostate adenocarcinoma datasets of patients segregated for low and high *C7* expression as indicated in the Oncoprints presented in Fig. S8c, d, respectively. Log-rank (Mantel–Cox) Test was performed to compare survival curves; *p < 0.05; ***p < 0.001. **k** Scatter plot showing the correlation of *C7* and *BRF1* mRNA expression in MSKCC (2010) prostate adenocarcinoma dataset (*n* = 150; all tumours with mRNA expression data). Pearson correlation coefficient and *p* value for the comparison is stated in the main text. **l** Schematic summary of key observations as described in the main text

We generated an in vitro model system by engineering PC3 clones to stably express eGFP-tagged *BRF1* (BRF1 CL4, 5, 6), or eGFP empty vector (Ctrl CL1, 2) as controls. Parental PC3 and CL1 and CL2 cells exhibited similar protein synthesis (Fig. S8a) and so CL2 was thereafter used as a representative control. Overexpression of BRF1 (Fig. 4a) resulted in significantly increased protein synthesis [*p*.adj = 0.0132 (CL4); 0.0024 (CL5); 0.0047 (CL6)] (Fig. 4b), demonstrating BRF1 expression promoted Pol III function as expected.

Stable isotope labelling by amino acids in cell culture followed by mass spectrometry (Fig. S8b) on culture media from BRF1 overexpressing PC3 cells revealed 33 common proteins which had > 1.2 fold change in abundance in the same direction in CL4, CL5 and 6 compared with Ctrl CL2 (Fig. 4c; Table S15). Reduced levels of complement factor D (CFD) in the secretome of BRF1 overexpressing cells was validated (Fig. 4d) and this was particularly interesting because the complement pathway, including *C7*, an ‘endpoint’ component of the MAC, was highlighted from RNA-Seq and proteomic data (Tables S10 and S14). Reduced *C7* mRNA (*p* = 0.0244) (Fig. 4e) and CFD and C7 protein expression (Fig. 4f) was validated in murine prostate tumours. Taken together, these data indicate that complement pathway activation is reduced in *Pten*^*Δ/Δ*^ *BRF1*^*Tg*^ tumours.

### Low C7 levels are clinically relevant in human prostate cancer

In clinical PCa cBioPortal data, alterations in *BRF1* and *C7* significantly co-occurred when interrogating all (*p* = 0.002) and metastatic (*p* < 0.0001) tumour cases in MSKCC (2010) dataset (Fig. 4g, h). Segregating cases revealed that patients with low *C7* expression (Fig. S8c, d) have a poorer prognosis in both datasets (Fig. 4i, j; Tables S16 and 17), in keeping with mouse model data. Furthermore, an inverse correlation between *BRF1* and *C7* mRNA expression and a positive correlation between *CFD* and *C7* mRNA expression was found in both MSKCC (2010) and TCGA (provisional) datasets (*BRF1* vs *C7*: Pearson correlation coefficient, *r* = −0.307; *p* = 0.0001 and *r* = −0.173; *p* = 0.0001, respectively; *C7* vs *CFD*: Pearson correlation coefficient, *r* = 0.490; *p* < 0.0001 and *r* = 0.495; *p* < 0.0001, respectively) (Figs. 4k,S8e–g).

Complement activation can enhance the differentiation and activation of T lymphocytes [12]. In both MSKCC (2010) and TCGA (provisional) datasets, a positive relationship between *C7* and the status of either *CD3E* [MSKCC: Pearson correlation coefficient, *r* = 0.324; *p* < 0.0001; TCGA: Pearson correlation coefficient, *r* = 0.139; *p* = 0.0019] or *CD4* [MSKCC: Pearson correlation coefficient, *r* = 0.356; *p* < 0.0001; TCGA: Pearson correlation coefficient, *r* = 0.205; *p* < 0.0001] (Fig. S8h–k) was observed, consistent with mouse model data.

In summary (Fig. 4l), *BRF1* expression is elevated in clinical PCa and its overexpression promotes prostate carcinogenesis in an established GEM model in vivo. Aberrant complement pathway activation, as indicated by altered secretion of CFD in vitro and reduced levels of CFD and C7 in vivo, is mechanistically consistent with the observed altered immune infiltrates, including neutrophils and CD4 positive T cells, within the tumour microenvironment upon elevation of *BRF1*.

## Discussion

For the first time, we show that BRF1 expression drives prostate carcinogenesis in vitro and in vivo. Recent work has highlighted that the repertoire of tRNAs expressed in oncogenic contexts specifically supports the production of proteins for a proliferative program [23]. Increases in specific tRNAs such as the initiator methionine tRNA^iMet^ are sufficient to promote migration and invasive behaviour in melanoma cells [24] and for fibroblasts to secrete collagen, which in turn enhanced tumour growth and angiogenesis [25], suggesting that BRF1 may play a major role in orchestrating altered programs of tRNA and thus protein synthesis. In support of this, we found that increased expression of *BRF1* upregulated global protein synthesis in vitro and mediated a switch in cellular programme, manifesting with an altered secretome.

To our knowledge, this is the first study to report effects of BRF1 on the immune response in an in vivo model. A previous study identified a direct effect of Pol III activity in enhancing macrophage phagocytic function in vitro [26]. Our study did not target Pol III activity directly in immune cells, but instead we show that elevated expression of BRF1 is associated with altered immune cell infiltration in an in vivo model of prostate carcinogenesis.

It is recognised that immune infiltration can have a significant influence upon clinical outcome in cancer patients [22]. Our previous study found that improved survival upon genetic loss of *Erk5* in *Pten*^*Δ/Δ*^ PCa model was related to increased levels of the T cell recruiting cytokines *Ccl5* and *Cxcl10* and enhanced T cell infiltration (predominantly CD4^+^) within the tumours [21]. Here, we report an association between reduced levels of CD4^+^ T lymphocytes, and also neutrophils, with poorer survival in *Pten*^*Δ/Δ*^ *BRF1*^*Tg*^ compared with *Pten*^*Δ/Δ*^ mice.

The complement pathway has been reported to have both pro- and anti-tumourigenic effects [11]. We observe that a low complement C7 environment is associated with poorer survival in our transgenic mouse model and human PCa. Furthermore, in both murine and human PCa, tumoral CD4^+^ T cell level positively correlated with complement C7 level. These data significantly contribute to our understanding of how the complement system may contribute to tumourigenesis.

In conclusion, our data highlight that elevated *BRF1* expression promotes prostate carcinogenesis. We implicate a non-cell autonomous role for high Brf1 expression in the prostatic epithelium as impacting on the tumour microenvironment with altered immune infiltrates. Given the rapid pace of development of cancer therapies targeting the immune system, further research in this area is warranted.

## Materials and methods

### Experimental methodology

Detail for the following experimental procedures is provided in Supplementary Materials and Methods: IHC; ISH; image analysis of IHC and ISH staining; histoscore; use and analysis of clinical datasets; culture of human PCa cell lines; transient transfection of plasmids and siRNA’s; generation of PC3 eGFP empty vector and eGFP-PRF1 stable clones; western blotting; WST-1 cell proliferation assay; cell cycle analysis from BrdU labelling; generation of conditional human BRF1 expressing mice; mouse strains and breeding; quantitative real-time PCR (Table S18); isolation and quantification of RNA (Table S19); RNA-sequencing and bioinformatic analysis; SILAC labelling and preparation of secretome samples; proteomics; comparison of RNA-Seq and proteomics data and ^35^S-Methionine incorporation assay.

### Human tissue microarray

Tissue microarrays (TMAs) consisting of cores of formalin-fixed, paraffin-embedded (FFPE) tissue from a cohort of 134 individuals with BPH and 516 patients with primary PCa were analysed for expression of BRF1 by immunohistochemistry. See Table S20 for demographic data at diagnosis: Age—median = 69.5 years (interquartile range (IQR) 63–75.5); Gleason sum score—median = 7 (IQR 6–8) (60.8% cases ≥ 7); Serum PSA levels—median = 15.55 ng/ml (IQR 7.025–44).

### Statistics

Statistical analyses, including: Mann–Whitney, Log-rank (Mantel–Cox), Pearson correlation coefficient, Welch’s *t* test (unpaired, 2 tailed) and Kaplan–Meier survival analysis, were performed using GraphPad Prism v7.02 or IBM SPSS Statistics 25 software. Bonferroni correction for multiple testing was performed as indicated. For all graphs, mean ± standard deviation (SD) or standard error of mean (SEM) (error bars) are presented and these are defined for each figure. In Forest plots, in both the upregulated and downregulated cases, the data were pooled (across all cell-lines), log-transformed, and a one-sample *t*-test was performed.

## Supplementary information

Supplementary Information

Table S10

Table S12

Table S14
