# Supplementary material for: BRF1 accelerates prostate tumourigenesis and perturbs immune infiltration
Source: Oncogene. 2019 Nov 18;39(8):1797–806. doi: 10.1038/s41388-019-1106-x (PMC7033044; doi:10.1038/s41388-019-1106-x)
Supplement: Supplementary file 1 — Supplementary Information [file 41388_2019_1106_MOESM1_ESM.docx]

**Supplementary:** BRF1 accelerates prostate tumourigenesis and perturbs immune infiltration.

**Running Title:** BRF1 driving prostate carcinogenesis.

**Authors:** Carolyn J. Loveridge^1,2,†^, Sarah Slater^2,†^, Kirsteen J. Campbell^1,2,†^, Noor A. Nam^2,#^, John Knight^2^, Imran Ahmad^1,2^, Ann Hedley^2^, Sergio Lilla^2^, Peter Repiscak^2^, Rachana Patel^2^, Mark Salji^1,2^, Janis Fleming^2^, Louise Mitchell^2^, Colin Nixon^2^, Douglas Strathdee^2^, Matthew Neilson^2^, Chara Ntala^1,2^, Sheila Bryson^2^, Sara Zanivan^1,2^, Joanne Edwards^1^, Craig N. Robson^3^, Carl S. Goodyear^4^, Karen Blyth^2^, and Hing Y. Leung^1,2*^

**Author affiliations**

^1^ Institute of Cancer Sciences, College of Medical, Veterinary and Life Sciences, University of Glasgow, Bearsden, Glasgow, G61 1QH, UK.

^2^ CRUK Beatson Institute, Bearsden, Glasgow, G61 1BD, UK.

^3^ Northern Institute for Cancer Research, The Medical School, Newcastle University, Framlington Place, Newcastle upon Tyne, NE2 4HH, UK.

^4^ Institute of Infection, Immunity and Inflammation, College of Medical, Veterinary and Life Sciences, University of Glasgow, Glasgow, G12 8TA, UK.

^†^ These authors contributed equally to the work.

* **To whom correspondence should be addressed:**

Prof. Hing Y. Leung, CRUK Beatson Institute, Bearsden, Glasgow, G61 1BD, UK, tel: 44(0) 141 330 3658, fax: 44(0) 141 942 6521, e-mail: h.leung@beatson.gla.ac.uk.

**Competing Interests:** the authors have nothing to disclose.

**Supplementary Materials and Methods**

**Immunohistochemistry (IHC)**

(i) Automated Staining

Immunohistochemistry (IHC) for BRF1, Ki67, Cleaved Caspase 3, p21, F4/80, NIMP, B220, CD138, CD3, CD4 and CD8 was performed on FFPE sections from human prostate core biopsy TMAs or murine prostate samples (n=minimum of 5 for each genotype) using Dako Autostainer (Brf1, Ki67, and CD138) or Leica Bond Rx Autostainer (Cleaved Caspase 3, p21, F4/80, NIMP, B220, CD3, CD4 and CD8) as previously described [1] with the exception of antigen retrieval conditions. The following buffers, temperatures and timings were used for antigen retrieval: pH6 (Thermo Fisher, TA-250-PM1X) at 98°C for 25 min; Enz 1 (Leica, AR9551) at 37°C for 10 min and ER2 (Leica, AR9640) at 98°C for 20 min.

Primary antibodies, dilutions and antigen retrieval conditions used were: BRF1 (Bethyl Laboratories Inc., IHC-00270, 1:50, pH6), Ki67 (SP6) (Thermo Fisher Scientific, RM-9106, 1:100, pH6), Cleaved Caspase-3 (Asp175) (Cell Signalling Technology, 9661; 1:500, ER2), p21 (Madrid (CNIO), mp21-cdkn1a, 1:10, ER2), F4/80 (Abcam, ab6640, 1:100, Enz 1), NIMP (Abcam, ab2557, 1:600, Enz1), B220 (Abcam, Ab64100, 1:200, ER2), CD138 (Sino Biological, 50641-RP02, 1:1000, pH6), CD3 (Abcam, ab16669, 1:100, ER2), CD4 (eBioscience, 14-9766-82, 1:500, ER2) and CD8 (4SM 15) (eBioscience, 14-0808-82, 1:500, ER2).

Dako Envision anti-rabbit secondary reagent (Agilent, K4003) was used in the case of BRF1, Ki67, Cleaved Caspase 3, CD138 and CD3 staining; Vector Laboratories ImmPRESS^™^ anti-Rat secondary reagent (MP-7444) and Intense R (Leica, DS9263) detection were used in the case of F4/80, NIMP, B220, CD4, CD8 and p21 staining.

(ii) Manual Staining

Manual IHC staining for FOXP3, CD21 and GL7 was performed on murine prostate samples (n=5 for each genotype) as previously described [1]. Primary antibodies used were: FOXP3 (Cell Signalling Technology, 12653, 1:100, pH6), CD21 (Abcam, ab75985, 1:250, pH6) and GL7 (Biolegend, 144602, 1:250; pH6). Dako Envision anti-rabbit secondary reagent (K4003) was used in the case of FOXP3 and C21 staining; Vector Laboratories ImmPRESS^™^ anti-Rat secondary reagent (MP-7444) was used in the case of B220 staining.

**In situ hybridization (ISH)**

Single ISH detection for *Cd19* (Advanced Cell Diagnostics (ACD) Probe: 314718), *Cd79a* (ACD Probe: 460188 and *Pax5* (ACD Probe: 311488) was performed using RNAscope® 2.5 LS reagent kit (brown) (ACD, 322100) on the Lecia Bond Rx Autostainer according to manufacturer’s instructions. For all single stains, positive staining was indicated by brown punctate dots present in the nucleus and/or cytoplasm.

**Image analysis of IHC and ISH staining**

Presented images from scanned ‘virtual’ slides were generated by exporting a field of view from SlidePath (Leica Biosystems) software or using the Figure Maker tool within HALO software.

Scoring of IHC staining was performed using algorithms designed within HALO software to quantitate % stained area in scanned slides (20x magnification) or probe copies per µm^2^ (ISH) (40x magnification). Prior to image analysis, representative regions of prostate tumour epithelium (Ki67 and cleaved caspase 3 IHC), all visible regions of stromal tissue (including immune cell aggregates) (F4/80, NIMP, CD3, CD4, CD8, B220 and FOXP3 IHC; *Cd19*, *Cd79a* and *Pax5* ISH) or just immune aggregates within the stroma (for H+E, CD138, CD21 and GL7 IHC) were manually annotated for all visible lobes on each slide. 5 slides per genotype being studied were assessed and all annotated regions on each slide were submitted for image analysis. The image analysis results for a batch of slides were exported to Microsoft Excel. The overall % positive stained area for each sample was calculated by dividing positive stained area by total annotated tissue area for each slide.

**Histoscore**

Evaluation of nuclear BRF1 IHC staining of human TMA core biopsies was performed by two individuals (NN and IA) using a weighted Histoscore method as previously described [2]. Briefly, nuclear BRF1 expression within total visible epithelial tissue on each core was scored at magnification × 40 by the two independent observers, who were blinded to clinical data. An interclass correlation coefficient of > 0.85 between the two scorers was achieved in all cases before formal analysis. The weighted histoscore method assesses the staining intensity and the percentage of cells stained with that intensity. It is calculated by (1 × % cells staining weakly positive)+(2 × % cells staining moderately positive)+(3 × % cells staining strongly positive). This provides a semi-quantitative classification of staining intensity, with the maximum score being 300 (if 100% of cells stain strongly positive) and minimum score being 0 (if 100% or cells are negative). The weighted histoscore method is a well-established method for scoring tissue that has heterogeneous staining. Median BRF1 histoscore for the PCa cohort was used as the cut-off point to assign cases as having low or high expression of BRF1 for survival analysis.

**Use and analysis of clinical datasets**

Progression-free survival and gene expression were queried using the cBioportal for cancer genomics platform [3, 4] in TCGA (provisional) and MSKCC (2010) [5] prostate adenocarcinoma datasets. Co-expression graphs were downloaded directly from cBioportal. Kaplan-Meier survival curves on extracted survival data were generated and statistical correlation of exported mRNA expression data was performed using GraphPad Prism v7.02 software.

**Culture of human prostate cancer cell lines**

Human prostate cancer (PCa) LNCaP, PC3, PC3M, DU145 cell lines were obtained from American Type Culture Collection (ATCC). LNCaP-AI cells were generated from LNCaP cells through chronic steroid deprivation over a period of 8 months to mimic androgen deprivation therapy [6]. LNCaP, PC3 and PC3M cells were grown in RPMI-1640 medium supplemented with 10% foetal bovine serum (FBS) and 2 mM L-glutamine; DU145 cells were grown in DMEM medium supplemented with 10% foetal bovine serum (FBS) and 2 mM L-glutamine; LNCaP-AI cells were grown in RPMI-1640 medium supplemented with 10% charcoal stripped serum (CSS) and 2 mM L-glutamine.

Cell lines were authenticated by LCG standards or in-house using Promega GenePrint® 10 Kit, according to the manufacturer’s instructions. All cell lines in the laboratory are routinely tested every 6 months for mycoplasma by LCG standards or in-house using Lonza MycoAlert™ Mycoplasma Detection Kit, according to the manufacturer’s instructions. The length of time between thawing of cells and their use in the experiments described was within 12 weeks.

**Transient transfection of plasmids and siRNA’s**

eGFP empty vector (eGFP-C1) was obtained from Takara Clontech. eGFP-*BRF1* construct has been used previously [7]. HA empty vector (pCDNA3-HA) was obtained from Invitrogen. HA-*BRF1* (pCDNA3-HA-*BRF1*) has been used previously [8, 9]. Control and human *BRF1* siRNA sequences are outlined in Table S19.

Human PCa cells were transfected for 48 hr with specified plasmids or siRNAs using Lipofectamine™ LTX reagent (Invitrogen) or Lipofectamine™ RNAiMAX (Invitrogen) respectively according to the manufacturer’s instructions prior to subsequent experimental analysis.

**Generation of PC3 eGFP empty vector and eGFP-PRF1 stable clones**

After transfection for 48 hr, PC3 cells stably expressing eGFP empty vector or eGFP-*BRF1* were selected using G418S sulphate solution (FORMEDIUM ™) (300 µg/mL).

**Western blotting**

Whole cell lysates (WCL) from PCa cell lines were prepared as previously described [1]. WCL from fine cryoground prostate tissue were obtained by homogenising prostates in 300 µL T-PER tissue protein extraction reagent (ThermoFisher Scientific), containing 1x protease inhibitor cocktail mix 1 (Calbiochem), 50 µg/mL phenylmethylsulfonyl fluoride (PMSF) and 1 mM dithiothreitol, using a Precellys tissue homogeniser. WCL were resolved by SDS-PAGE on 10% or 4-12% gradient polyacrylamide gels (Invitrogen) at 180 V for 1 hr and transferred electrophoretically using a wet transfer system onto PVDF membrane (Milipore) at 100 V for 1 hr. Blots were blocked for 1 hr with 5% skimmed milk, rinsed and probed with the required primary antibody (diluted in 5% BSA, 0.1% Tween-20 containing TBS and 0.05% sodium azide) overnight at 4°C. Following incubation with appropriate HRP conjugated secondary antibody [anti-rabbit IgG (Cell Signalling, 7074) or anti-mouse IgG (Cell Signalling, 7076)] (diluted 1:5000 in 5% skimmed milk), bands were visualised using X-ray film or BioRad ChemiDoc™ gel imaging system and software. Primary antibodies used were: BRF1 (Bethyl Laboratories, Inc., A301-228A, 1:5000), Beta Actin (Santa Cruz Biotechnology, sc-1615, 1:1000), Alpha Tubulin (Sigma, T6557, 1:2000), HSP70 (Abcam, Ab3148-500, 1:5000), CFD (Santa Cruz Biotechnology; sc-376015, 1:10000), C7 (Abcam, ab192346, 1:1000), HSP70 (Abcam, Ab3148-500, 1:5000) and HSC70 (Santa Cruz Biotechnology, sc-7298, 1:1000).

**WST-1 assay**

PCa cells were seeded in 96 well plates in a 0.1 mL volume, transfected with stated plasmids or siRNAs for 48 hr then 10 μl of water soluble tetrazolium salt-1 (WST-1) (Roche) reagent was added to cells. After incubation for 120 min at 37°C and 5% CO_2_, the absorbance of the samples at 450 nm and the reference wavelength, 650 nm was measured using a microplate reader.

**Cell cycle analysis from BrdU labelling**

Cells were pulse labelled with BrdU (Cell labelling reagent, VWR; 1:1000) for 1 hour prior to harvesting and fixation in 70% ethanol at -20°C for at least 1 hour. Cells were then washed in PBS prior to incubation in 100 μl PBS and 100 μl 4 M hydrochloric acid for 15 min at room temperature. Cells were next washed with phosphate buffered saline (PBS) then PBST [PBS containing 0.5% Tween 20 and 1% bovine serum albumin (BSA)] prior to incubation with anti-BrdU antibody (BD Biosciences, 347580; 1:40 in PBST) for 30 min at room temperature. Cells were then washed with PBST prior to incubation with anti-mouse Alexa Fluor 488 secondary antibody (Thermo Fisher Scientific; A-11001; 1:40 dilution in PBST) for 30 min at room temperature in the dark. After washing with PBST then PBS, cells were finally resuspended in 300 μl PBS containing 10 μg/ml Propidium Iodide for 30 min at room temperature prior to analysis on a FACS Calibur machine.

**Generation of conditional human *BRF1* expressing mice**

Conditional human *BRF1* expressing mice were generated by targeting a lox-stop-lox transgene under the control of a CAG promoter to the *Hprt* locus [10]. The CAG lox-stop-lox transgene was constructed by firstly replacing the splice acceptor of plasmid pBigT with the CAG promoter from pTurbo-Cre (gift from Prof. Tim Ley). The PGK promoter and the EM7 promoter sequence from pL452 were subsequently inserted into pBigT-CAAG by recombineering in *E. coli*. Short 5’ and 3’ arms homologous to sequences within the *Hprt* targeting plasmid pSKB1 were then inserted up and downstream of the expression cassette to generate p*Hprt*.CAAG.STOP. A cDNA encoding the LifeAct-mEGFP fusion protein was then cloned downstream of the lox-stop-lox. The transgene was then recombined into pSKB1 to generate the final targeting vector.

*Hprt*-deficient HM1 ES cells were electroporated with linearised vector then cells were subsequently cultured on a DR4 mouse embryonic fibroblast feeder layer [11]. Homologous recombinants were selected in medium containing HAT supplement (Sigma). Correct targeting of the vector to the *Hprt* locus on both the 5’ and 3’ sides was confirmed using PCR on genomic DNA prepared from HAT-resistant colonies. PCR genotyping was done using Expand Long Template (Roche) according to the manufacturer's recommendations. Primers used for genotyping targeted ES cells were 5′ GTTGCTGAGGCAAAAATAGTGTAAT and CCATTTACCGTAAGTTATGTAACGC and 3’ CTACCTAGTGAGCCTGCAAACTG and ATGTAAGTGCTAGGAATTGAACCTG.

Following identification of correctly targeted clones, mouse lines were derived by injecting ES cells into C57BL/6J blastocysts according to standard protocols [12]. Germline transmission was identified by coat colour and correct transmission of the transgene was confirmed by PCR amplification of the *BRF1* sequences using the primers 5´ GCGAATGCATCTCCTCTCAG and 3´ ACACTGGTCACAGGGTCAGC.

**Mouse strains and breeding**

All small animal (murine) experiments were approved by the Animal Welfare and Ethical Review Board (AWERB) at the University of Glasgow and performed under UK Home Office licence numbers 30/3185 and P5EE22AEE in accordance with relevant guidelines and regulations (EU directive 2010/Animal (Scientific Procedures) Act 1986). Mice were housed in conventional cages under a constant 12-hour light/dark cycle and given access to standard diet and water *ad libitum*. Mice were maintained on a mixed background (C57BL/6J) and were genotyped by Transnetyx^TM^. *BRF1^Tg^* mice were born at the expected Mendelian ratio and were phenotypically indistinguishable from littermate controls. Male mice were allocated to cohorts based upon specific genotypes and strain-matched male littermates (Cre expressing mice with no other genetic alterations or mice not expressing Cre) were used as controls. Mice were handled, monitored for PCa development and aged to ethically approved clinical end points. Assessment of clinical end point was based upon mice having a palpable tumour and obvious swollen abdomen.

**Isolation and Quantitation of RNA**

RNA was isolated using RNeasy Mini Kit (Qiagen) as per manufacturer’s instructions including a DNase treatment step. RNA samples were quantified by spectrophotometry using a NanoDrop 2000 spectrophotometer (Thermo Scientific). For RNA-Sequencing (RNA-Seq) samples only, the quality of isolated RNA was assessed by running the samples on a 2100 Bioanalyzer (Agilent) to generate RNA electropherograms, and obtain RNA integrity number (RIN) (range 7-10).

**Quantitative real time PCR**

First strand cDNA was prepared using High Capacity cDNA Transcription Kit (Applied Biosystems) according to manufacturer’s instructions and qPCR was performed using the Taqman technique as previously described [1]. Specific primer/probe combinations are outlined in Table S20.

**RNA sequencing and bioinformatic analysis**

RNA was isolated from *Pten^Δ/Δ^* and *Pten^Δ/Δ^* *BRF1^Tg^* cryoground prostate tissue, quantified and its quality assessed as outlined above. Libraries from these samples were prepared for sequencing using the Illumina™ TotalPrep™ RNA Amplification Kit (Ambion, Life Technologies, AMIL1791) with Poly(A) selection according to the manufacturer’s instructions. Amplified libraries were sequenced by Beijing Genomics Institute (BGI). Prior to sequencing, the average fragment length was quantitated using a Agilent 2100 Bioanalyzer with Agilent DNA 100 reagents and the RNA yield was quantitated by qPCR to validate quality of library preparation. TruSeq PE Cluster Kit V3-cBot-HS (Illumina) was used for paired end cluster generation. The library was pair end sequenced on the Illumina® HiSeq 2000 platform, with 90 nucleotide reads and at least 6 Gb of reads generated per sample.

Quality checks on the raw RNA-Seq data files were done using fastqc version 0.10.1 (http://www.bioinformatics.bbsrc.ac.uk/projects/fastqc) and fastq_screen version 0.4.2 (http://www.bioinformatics.babraham.ac.uk/projects/fastq_screen/). RNA-Seq reads were aligned to the GRCm38 [13] version of the mouse genome using tophat2 version 2.0.10 [14] with Bowtie version 2.1.0.0 [15]. Expression levels were determined and statistically analysed by a combination of HTSeq version 0.9.1 (http://www-huber.embl.de/users/anders/HTSeq/doc/overview.html), the R environment, version 3.1.1, utilizing packages from the Bioconductor data analysis suite and differential gene expression analysis based on the negative binomial distribution using DESeq [16]. Pathway analysis of genes with an absolute fold change > 1.5 and an adjusted P value < 0.05 was performed using GeneGo Pathways Software (MetaCore; https://portal.genego.com/version 6.35.69300).

**SILAC labelling**

PC3 stable clones (Ctrl. CL2, BRF1 CL4, 5 and 6) were each cultured for approximately 6-8 weeks in RPMI 1640 media for SILAC (Thermo Scientific) containing 10% dialysed foetal bovine serum (GIBCO), 2 mM L-glutamine, G418 (300 µg/mL) and the following amino acids: Lysine 4 (48 µg/mL) and Arginine 6 (200 µg/mL) (for ‘Medium’ label) or Lysine 8 (48 µg/mL) and Arginine 10 (200 µg/mL) (for ‘Heavy’ label). Incorporation of the labels in each PC3 clone was checked prior to preparation of secretome samples.

**Preparation of secretome samples**

PC3 stable clones (Ctrl. CL2, BRF1 CL4, 5 and 6) were seeded in 10 cm plates until 70-80% confluency then washed three times with PBS before incubating in serum free RPMI 1640 medium containing 2 mM L-glutamine and G418 (300 µg/mL) for 72 hr. Conditioned media were then collected and subjected to three rounds of 10 min centrifugations at increasing speeds (300 x *g* followed by 2000 x *g* followed by 10,000 x *g*) at 4°C to ensure removal of cellular debris. Supernatant was then acidified to pH7.5 using trifluoroacetic acid prior to the addition of StrataClean beads (Agilent) (10 µL/mL). Samples were incubated for 1 hr in a rotor wheel (40 rpm) at room temperature then beads were collected by centrifugation at 872 x *g* for 1 min and supernatant discarded. 4x NuPAGE® LSD Sample Buffer (Invitrogen) containing 0.1M DTT was added to collected beads prior to boiling at 95°C for 5 min. Samples were then centrifuged at 376 x *g* for 1 min and supernatants collected for further analysis.

**Proteomics**

**(i) Sample preparation.**

**Mouse prostate tumour samples – protein liquid digestion**

Cryoground mouse prostate tumour tissue samples were lysed in buffer containing: 4% SDS, 0.1M DTT, 0.1M Tris-HCL at pH = 7.4, without protease inhibitors. Reduced proteins were alkylated using 55mM Iodoacetamide for one hour at room temperature. Alkylated proteins were then precipitated in two steps: using 24% and 10% solutions of Trichloroacetic acid (TCA). In both steps, pellets were incubated at 4°C for 10 min and centrifuged at 14,000 rpm for 5 min. Supernatants were carefully aspirated, and pellets were finally washed with water until the supernatant reached neutral pH. Pellets were reconstituted in in 8 M urea solution and submitted to a two-step digestion. First using Endoproteinase Lys-C (Alpha Laboratories) for 1 hour at 35°C, after which partial digests were further digested, with trypsin (Promega) overnight at 35°C.

**Secretome samples – proteolytic digestion of proteins ‘in gel’**

Eluates from Strataclean beads were separated by SDS-PAGE, stained with Coomassie blue. Each gel lane was divided in 5 slices and digested according to a procedure previously described [17].

**(ii) Mass spectrometry analysis**

**Liquid chromatography**

Digested peptides were desalted using StageTip [18] and separated by nanoscale C18 reverse-phase liquid chromatography performed on an EASY-nLC II (Thermo Scientific) coupled to a Linear Trap Quadrupole - Orbitrap Elite (secretome samples) or Orbitrap Velos (mouse tumour samples) mass spectrometer (Thermo Scientific).

Elution was carried out using a binary gradient with buffer A: water and B: 80% acetonitrile, both containing 0.1% of formic acid.

Peptide mixtures were separated at 200 nl/min flow, using a 20 cm fused silica emitter (New Objective) packed in-house with ReproSil-Pur C18-AQ, 1.9μm resin (Dr Maisch GmbH). Packed emitter was kept at 35°C by means of a column oven integrated into the nanoelectrospray ion source (Sonation).

The gradient used start at 2% of buffer B, kept at same percentage for 5 min, then increased to 30% over 90 min and then to 60% over 15 min. Finally, a column wash was performed ramping to 80% of B in 5 min and then to 95% of B in one minute followed by a 13 min re-equilibration at 2% B for a total duration of 129 min. The eluting peptide solutions were automatically (online) electrosprayed into the mass spectrometer via a nanoelectrospray ion source (Sonation). An Active Background Ion Reduction Device (ABIRD) was used to decrease ambient contaminant signal level.

**(iii) Data dependent acquisition**

General mass spectrometric conditions of Linear Trap Quadrupole - Orbitrap were as follows: spray voltage, 2.1 kV (secretome samples) or 2.4 kV (mouse prostate tumour samples), ion transfer tube temperature, 200°C. The mass spectrometer was operated in positive ion mode and used in data-dependent acquisition mode (DDA). A full scan (FT-MS) was acquired at a target value of 1,000,000 ions with resolution R = 120,000 (secretome samples) or R = 60,000 (mouse prostate tumour samples) at 400 m/z, over mass range of 300-1650 amu (secretome samples) or 350-1600 amu (mouse prostate tumour samples). For secretome samples the top ten most intense ions were selected for fragmentation in the linear ion trap using higher energy collision dissociation (HCD) using a maximum injection time of 150 ms or a target value of 40000 ions. For mouse tumour samples the fragmentation was performed in the linear ion trap using Collision Induced Dissociation using the top ten most intense ions, a maximum injection time of 25 ms or a target value of 5000 ions.

Multiply charged ions from two to five charges having intensity greater than 40000 counts (secretome samples) or 5000 counts (mouse prostate tumour samples) were selected and fragmented using normalized collision energy of 30 (secretome samples) or 36 (mouse prostate tumour samples). Former target ions selected for MS/MS were dynamically excluded for 60 secs (secretome samples) or 25 secs (mouse prostate tumour samples).

**(iv) Data analysis**

The MS Raw files were processed with MaxQuant software version 1.5.5.1 and searched with Andromeda search engine, querying UniProt Homo sapiens (09/07/2016; 92,939 entries). The database was searched requiring specificity for trypsin cleavage and allowing maximum two missed cleavages. Methionine oxidation and N-terminal acetylation were specified as variable modifications, and Cysteine carbamidomethylation as fixed modification. The peptide, protein and site false discovery rate (FDR) was set to 1 %.

For quantitation of secretome samples in MaxQuant, multiplicity was set to 2 and Arg0/Arg10, Lys0/Lys8 were used for ratio measurement of SILAC labelled peptides. Only unique peptides were used for protein group quantification.

Proteins contained in mouse tumour samples were quantified according to the label-free quantification algorithm available in MaxQuant and the output from this was further processed and analysed using Perseus software version 1.5.5.3. The common reverse and contaminant hits (as defined in MaxQuant output) were removed. Only protein groups identified with at least one uniquely assigned peptide were used for the analysis. Significantly enriched proteins were selected using a two -sided t-test analysis with a 5% FDR. Two-dimensional Principal Component Analysis plots were generated using Perseus. From principal component analysis (PCA) of the data, it became clear that one *Pten^Δ/Δ^* and two *Pten^Δ/Δ^* *BRF1^Tg^* samples were outliers and did not segregate with other samples from the same genotype. Data from these samples were therefore not included in subsequent analysis.

The “Significance B” algorithm in Perseus was used to define which proteins were changing significantly in the Secretome samples.

**Comparison of RNA-Seq and proteomics data**

An expanded list of majority protein IDs identified from the proteomics analysis was mapped to Ensembl IDs (GRCm38.p6, v.93) using biomaRt package. In the case of duplicated mappings only genes with the smallest p.adj were kept for further analysis. Similarly, Ensembl IDs from the RNA-seq analysis were re-annotated against the same version of the database as used in the case of the proteomics data.

Further, lists of significantly (p.adj < 0.05 and absolute fold-change > 1.5) altered genes were generated to perform pathway enrichment analysis in GeneGO MetaCore (v.6.35 build 69300, https://portal.genego.com) from Thomson Reuters. These genes were further annotated with GOslim terms using biomaRt and genes with "immune system process" GO term (GO:0002376) are shown in RNA-seq heatmap and highlighted in proteomics volcano plot.

**^35^S-Methionine incorporation assay**

PC3 stable clones (Ctrl. CL1,2 and BRF1 CL4, 5 and 6) were grown to ~70% confluence, treated with ^35^S-methionine (Perkin Elmer) for 30 min at a final concentration of 30 μCi then protein synthesis was determined as previously described [19].

**Supplementary Figures**

**
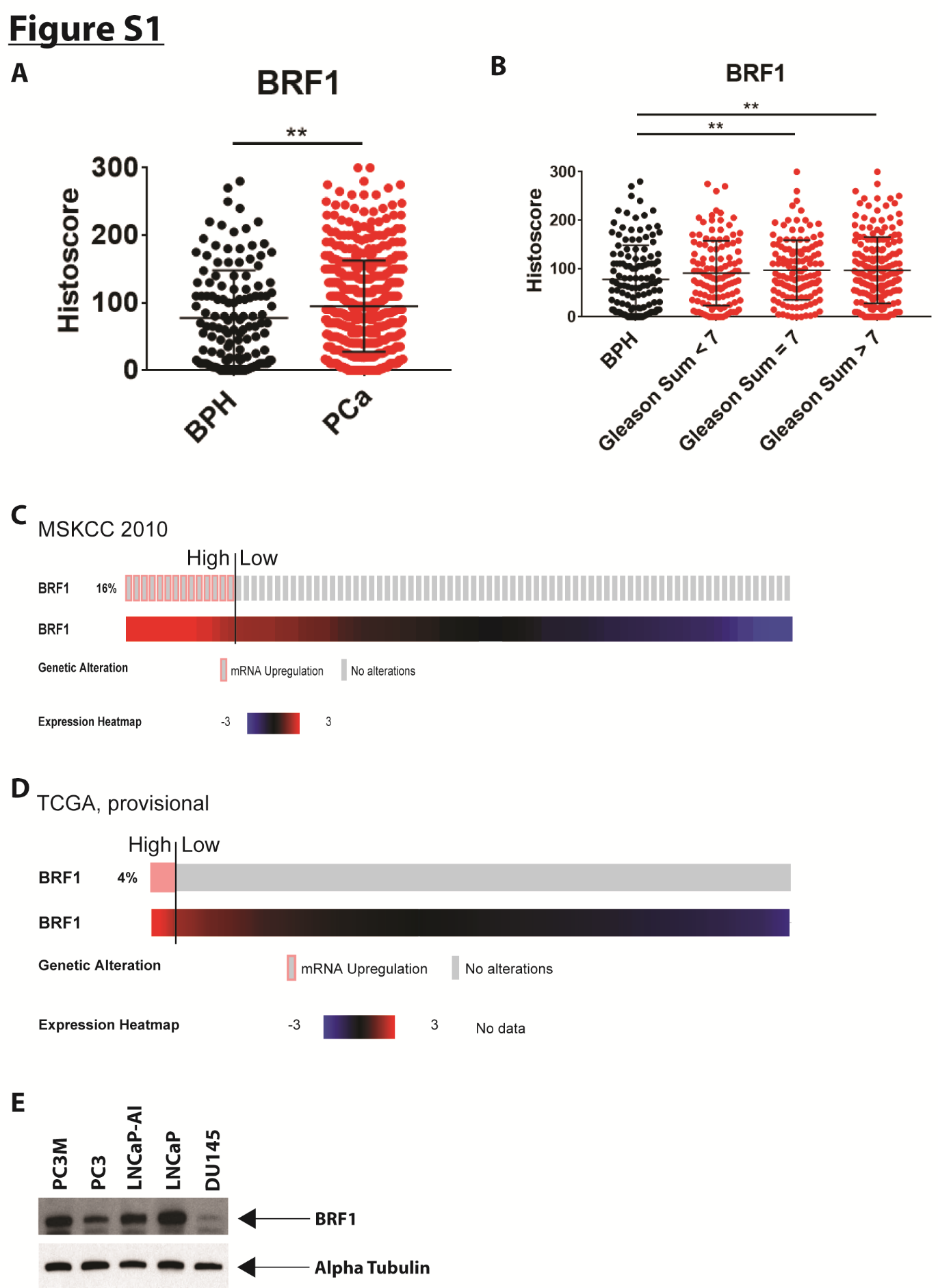
**


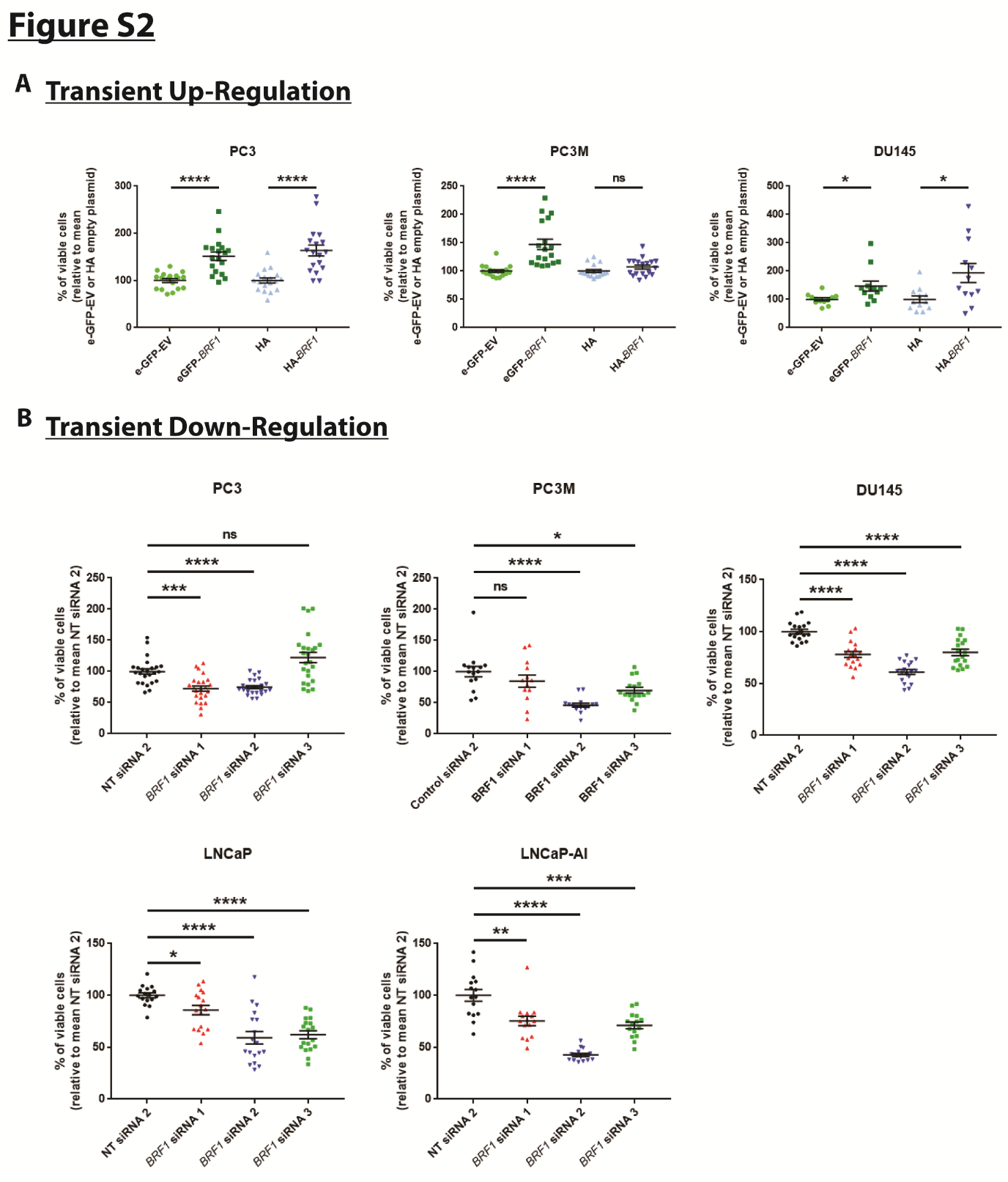


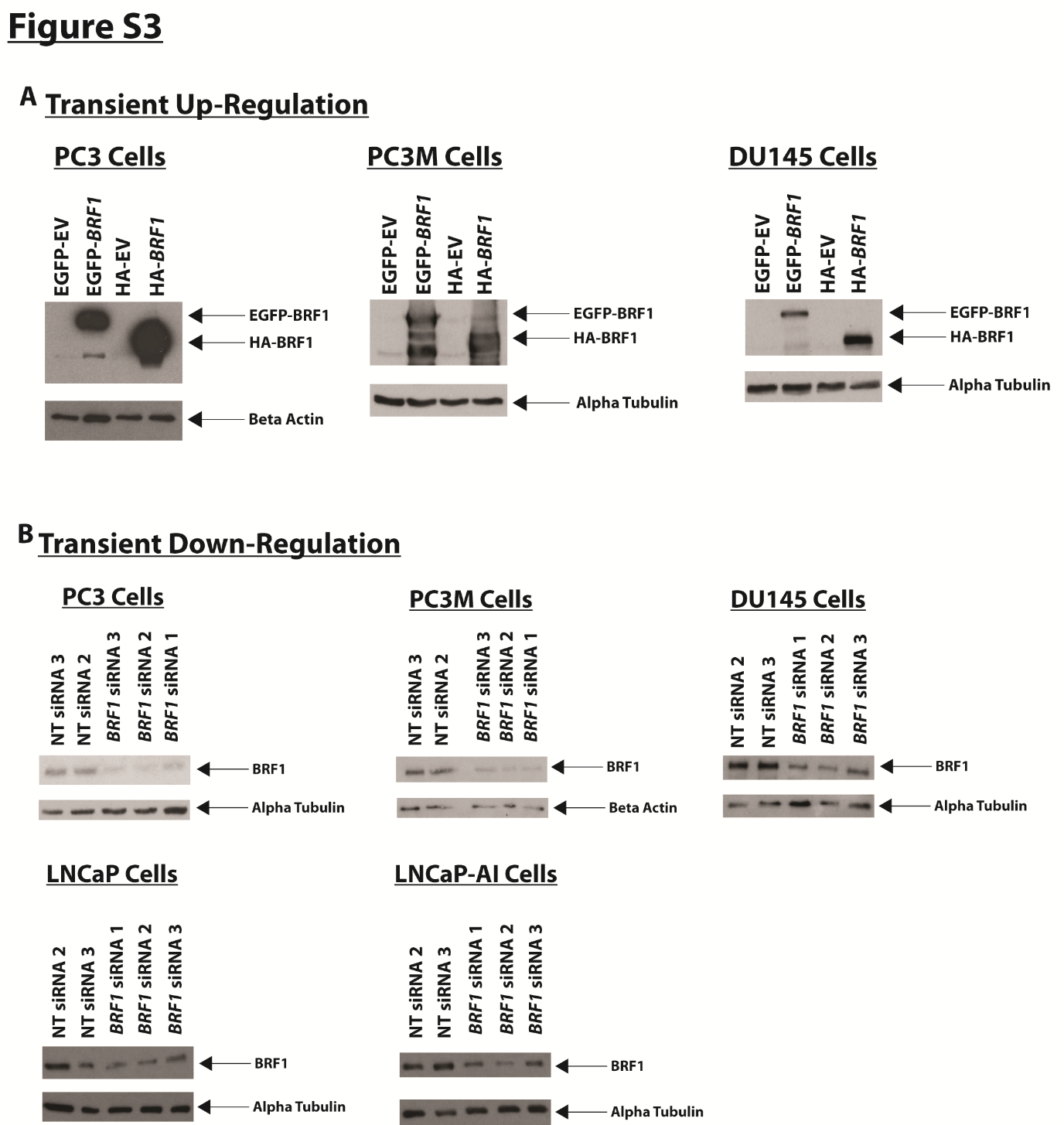


**
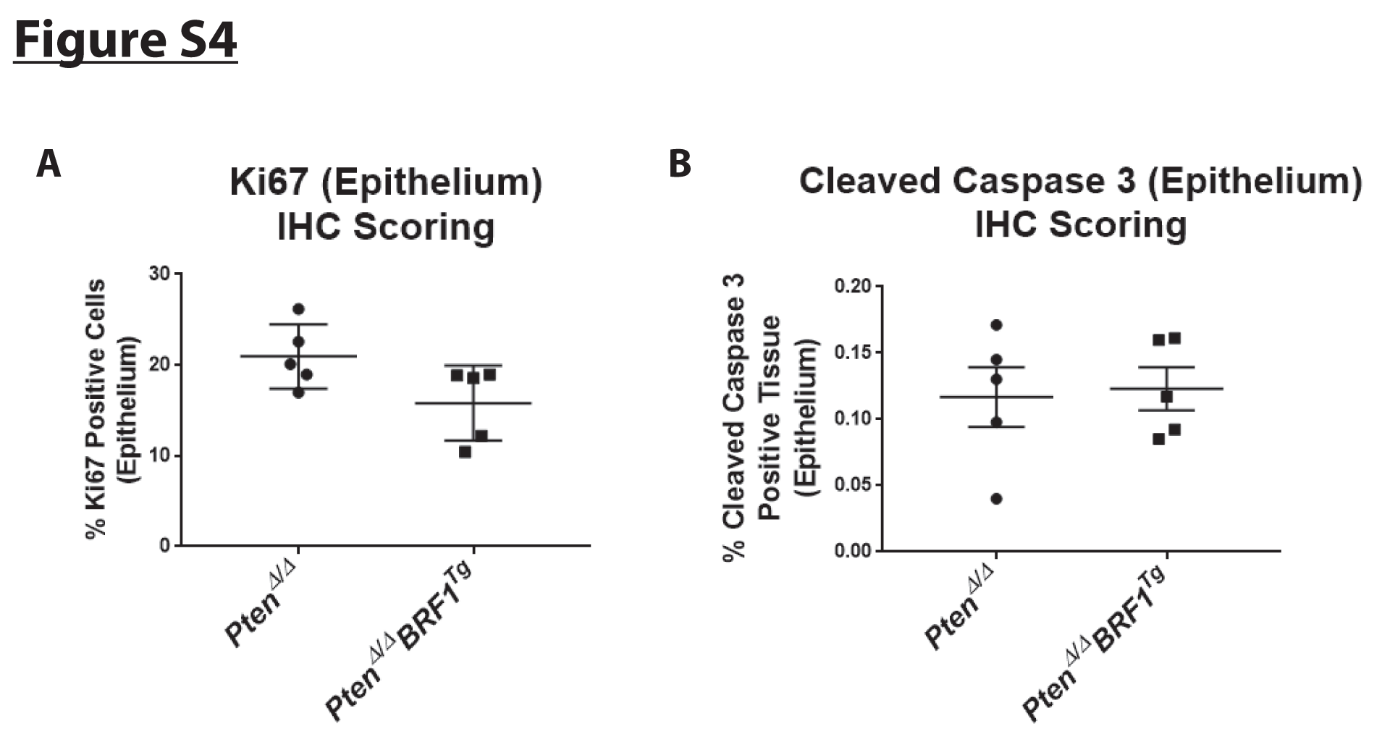
**


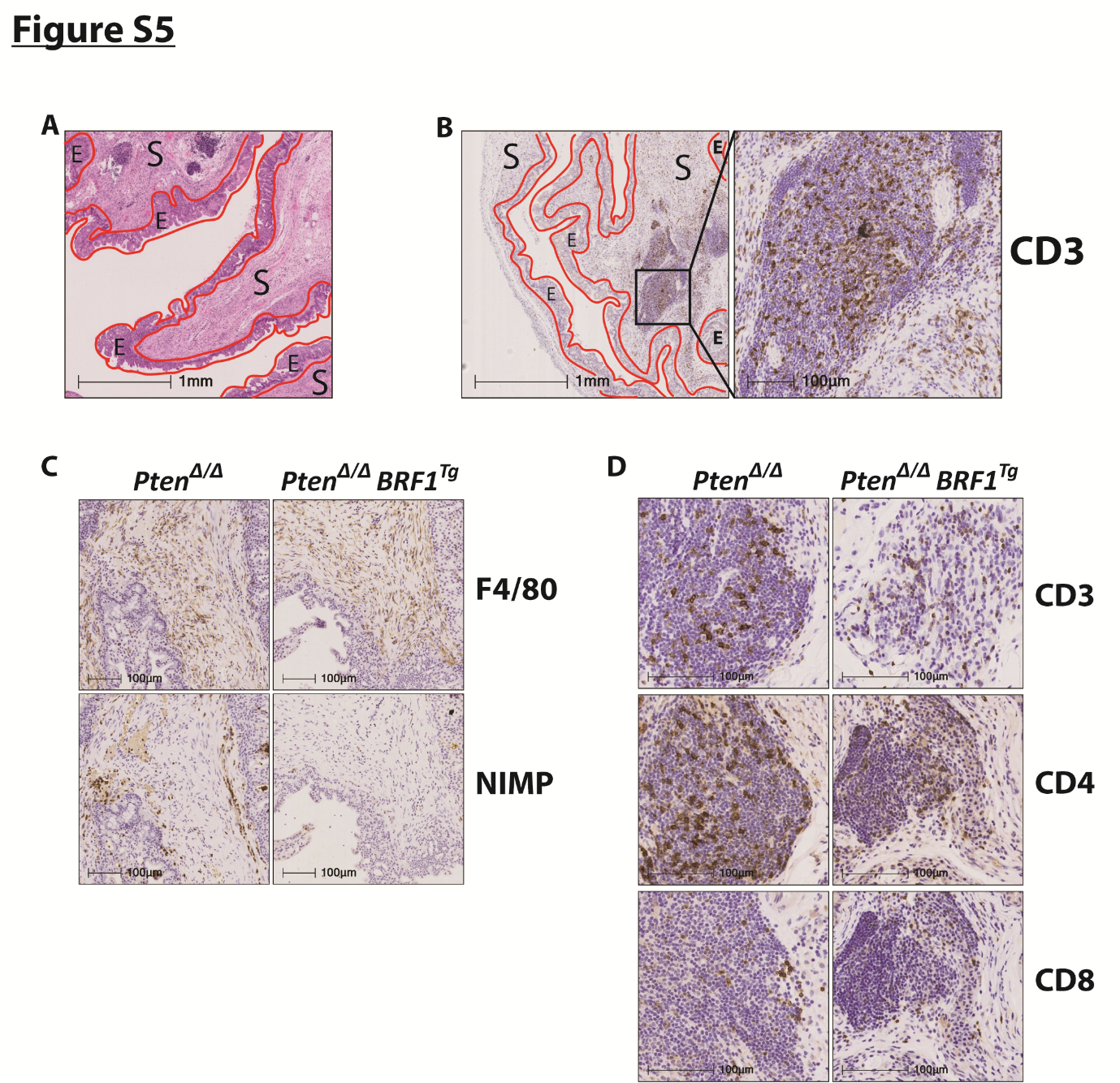


**
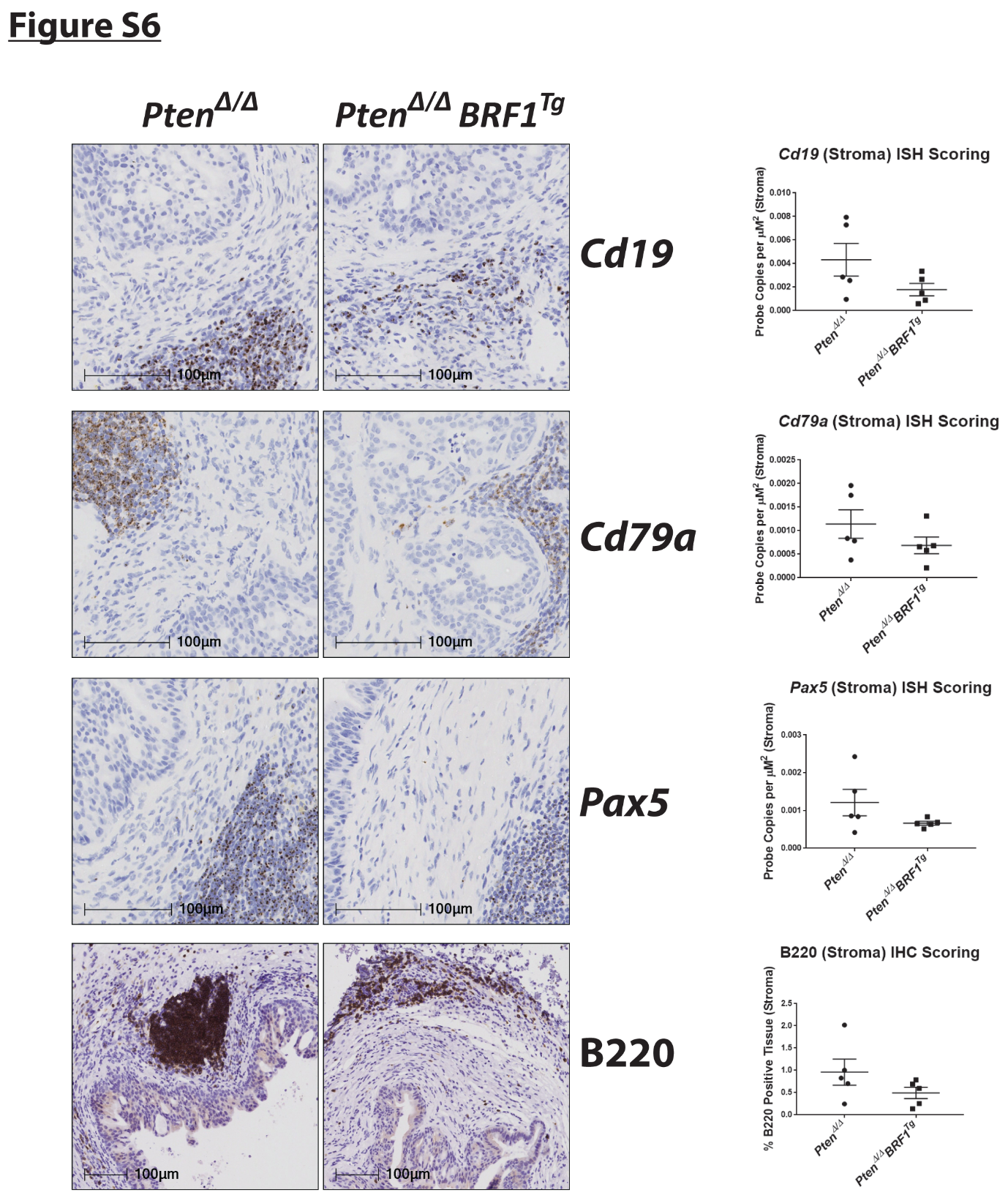
**


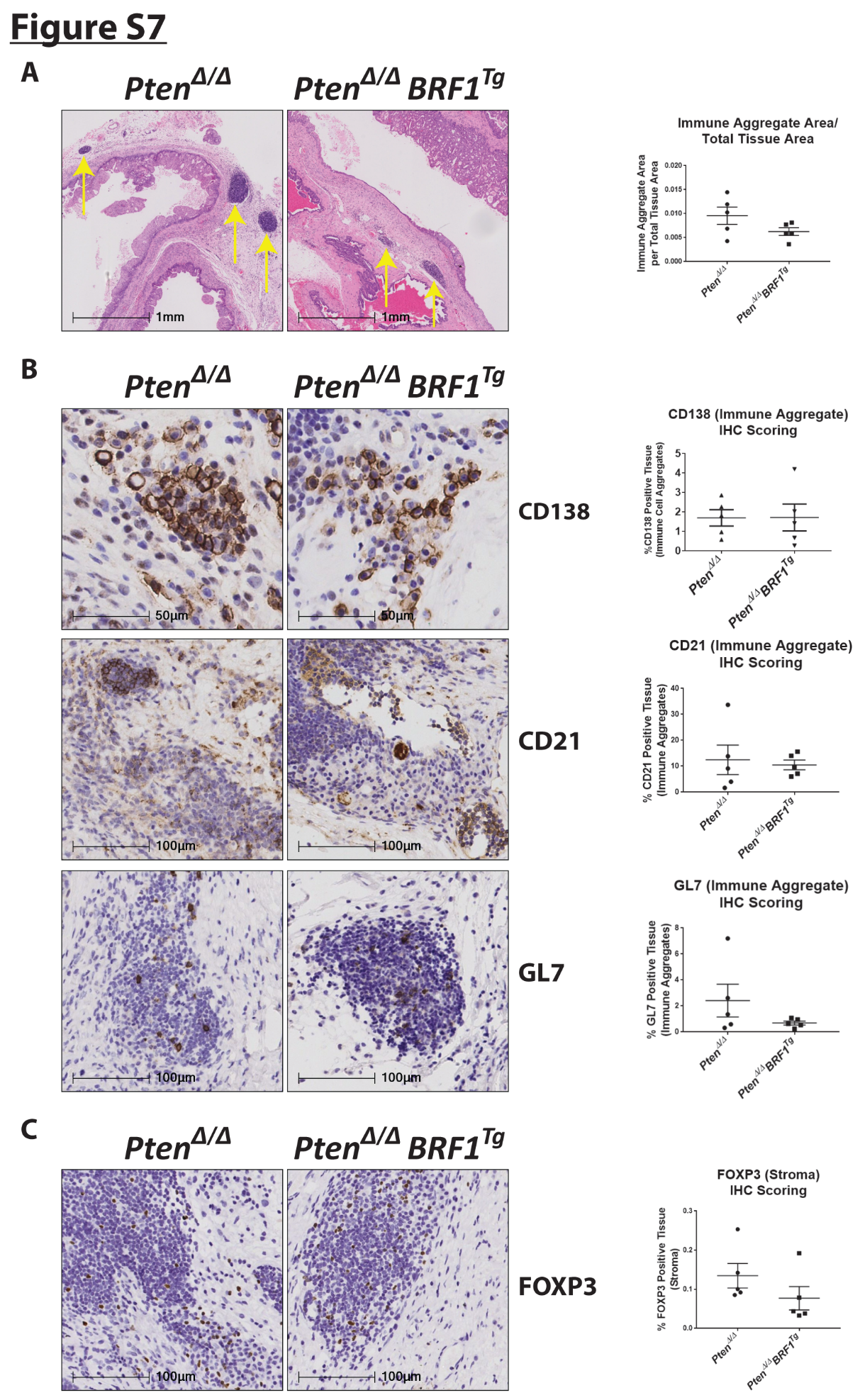


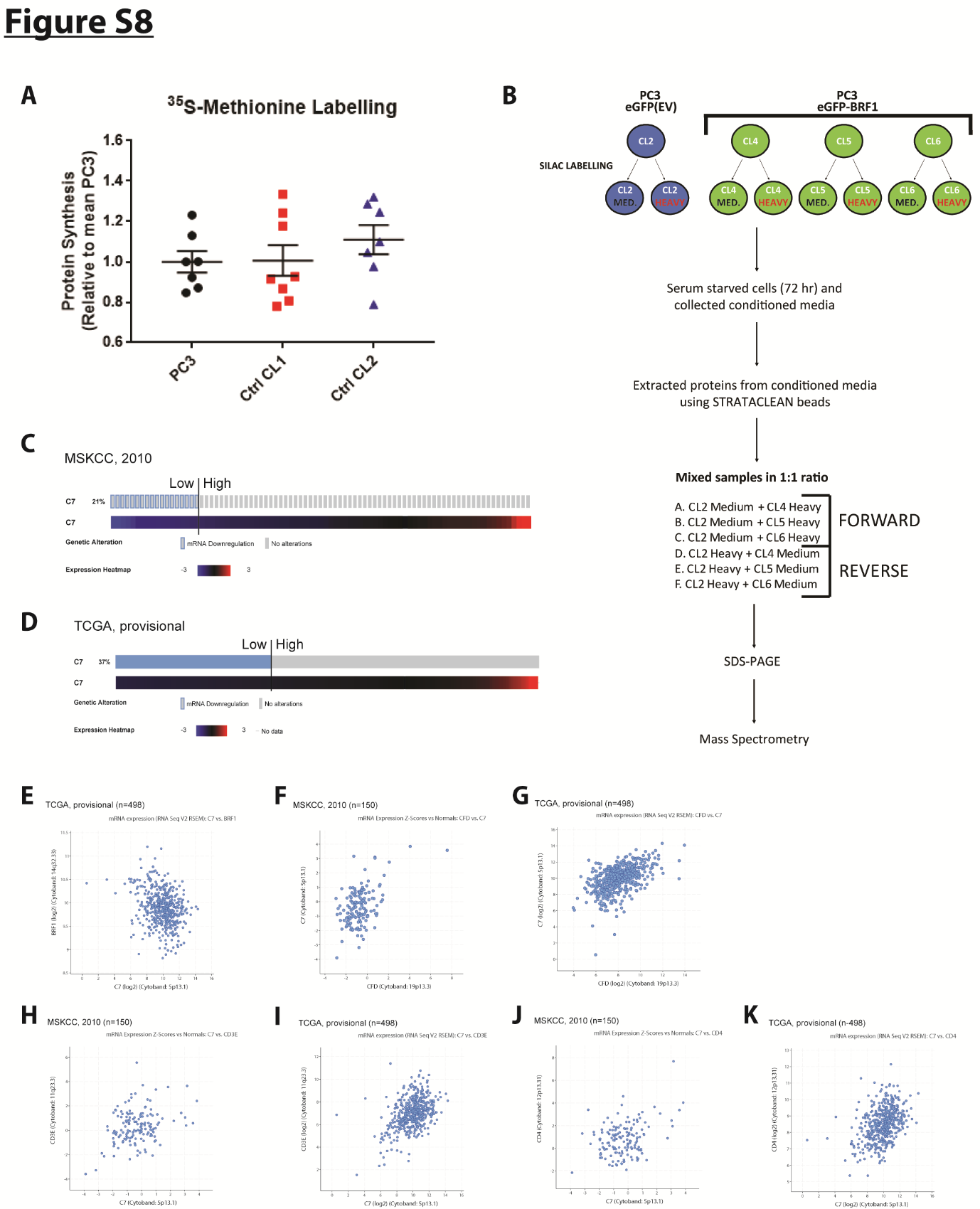


**Supplementary Figure Legends**

**Figure S1: BRF1 expression is elevated in PCa compared to BPH and is expressed in a panel of PCa cell lines.**

(**A**-**B**) The relationship between the mean histoscores of BRF1 expression was compared between BPH (n = 134) and PCa (n = 516) cohort as a whole (**A**) and between BPH with specific Gleason Sum score [< 7 (n=120), 7 (n=127) and > 7 (n=187)] within the PCa cohort (**B**). Individual data points are shown in presented graphs; horizontal line represents the mean; error bars represent standard deviation (SD). Mann-Whitney t test was performed; *:p < 0.05; **:p < 0.01; ***:p < 0.001. (**C**) Oncoprint and heat map of BRF1 expression in MSKCC (2010) dataset. Patients were divided into low and high BRF1 expressors based on z score > 2 for survival analysis presented in Figure 1C. (**D**) Oncoprint and heat map of *BRF1* expression in the TCGA (provisional) PCa dataset. Patients were divided into low and high *BRF1* expressors based on z score > 2 for survival analysis presented in Figure 1D. (**E**) Western blot of whole cell lysates prepared from a panel of PCa cell lines comprising: PC3M, PC3, LNCaP-AI, LNCaP and DU145 using an anti-BRF1 antibody. Alpha tubulin served as a loading control. Blot shown is representative of 3 independent experiments. Full blots are shown in Figure S7.

**Figure S2: Transient overexpression of *BRF1* increases cell proliferation whereas transient down-regulation of *BRF1* using siRNA reduces cell proliferation.**

(**A**-**B**) Analysis of cell proliferation in PC3, PC3M and DU145 cells which were transiently transfected with HA-*BRF1*, GFP-*BRF1* or their respective HA- and GFP-empty vector controls for 48 hr (in 4-6 wells per cell line per condition) (n=3 independent experiments) (**A**) and PC3, PC3M, DU145, LNCaP and LNCaP AI cells which were transiently transfected with 3 independent siRNA’s for BRF1 or control non-targeting (NT) siRNA 2 for 48hr (in 3-6 wells per cell line per condition) [n=3 (PC3M, DU145, LNCaP, LNCaP-AI cells) or n=4 (PC3 cells) independent experiments] (**B**) by WST1 cell proliferation assay. In (**A**), data for *BRF1* overexpressing constructs was normalised to mean of respective empty vector. Individual data points are shown in the presented graphs; long horizontal line indicates the Mean; error bars represent standard error of mean (SEM); Welch’s t test (unpaired, 2 tailed) was used to calculate p values; *: p < 0.05; ****: p < 0.0001; ns = not significant. In (**B**), data for *BRF1* siRNA’s was normalised to mean of NT siRNA 2. Individual data points are shown in the presented graphs; long horizontal line indicates the Mean; error bars represent SEM; Welch’s t test (unpaired, 2 tailed) with Bonferroni correction for multiple testing was used to calculate p.adj values; *: p.adj < 0.05; **: p.adj < 0.01; ***: p.adj < 0.001; ****: p.adj < 0.0001; ns = not significant.

**Figure S3: Confirmation of transient up- and down-regulation of *BRF1* expression.**

(**A**) Western blotting of whole cell lysates prepared from PC3, PC3M and DU145 cells which had been transiently transfected with HA-BRF1, GFP-BRF1 and their respective HA- and GFP-empty vector controls for 48 hr using an anti-BRF1 antibody. Beta actin (PC3 cells) and Alpha tubulin (PC3M and DU145 cells) served as loading controls. Blots shown are representative of 3 independent experiments for each cell line. (**B**) Western blotting of whole cell lysates prepared from PC3, PC3M, DU145, LNCaP and LNCaP-AI cells which had been transiently transfected with 3 independent siRNA’s for *BRF1* and two different control non-targeting (NT) siRNA’s for 48 hr using anti-BRF1 antibody. Alpha tubulin (PC3, DU145, LNCaP and LNCaP-AI cells) and Beta actin (PC3M cells) served as loading controls. Blots shown are representative of 3 independent experiments for each cell line.

**Figure S4: Ki67 and Cleaved Caspase 3 levels are not significantly changed in *Pten^Δ/Δ^ BRF1^Tg^* tumours**

(**A-B**) Scoring of Ki67 (**A**) and cleaved caspase 3 (**B**) IHC staining. Ki67 and cleaved caspase 3 IHC staining were analysed in 25 manually annotated areas of prostate tumour epithelium per slide from *Pten^Δ/Δ^* (n=5) and *Pten^Δ/Δ^* *BRF1^Tg^* (n=5) mice using HALO software (see methods). In (**A**-**B)**, individual data points are shown in the presented graphs; long horizontal lines indicate the Mean; error bars represent SEM.

**Figure S5: Analysis of Infiltration of neutrophils and CD4 positive T lymphocytes in *Pten^Δ/Δ^ BRF1^Tg^* and *Pten^Δ/Δ^* mice.**

(**A**) Representative micrograph of H+E staining in anterior prostate tissue from an end point *Pten^Δ/Δ^* sample with annotations to highlight epithelial (E; annotated with red line) and stromal (S) compartments. Scale bar is shown (1 mm). (**B**) CD3 IHC staining in anterior prostate tissue from an end point *Pten^Δ/Δ^* sample with annotations to highlight epithelial (E; annotated with red line) and stromal (S) compartments. Higher magnification image (right panel) of area highlighted by black box in lower magnification image (left panel) highlights tertiary lymphoid aggregate structures within the stroma that have positive staining for CD3. Scale bars are shown (1 mm in left panel; 100 µm in right panel). (**C**) Representative micrographs of F4/80 and NIMP IHC staining in anterior prostate tumour tissue from *Pten^Δ/Δ^* and *Pten^Δ/Δ^ BRF1^Tg^* mice (n=5 for each genotype). Scale bars are shown (100 µm for all images). (**D**) Representative micrographs of CD3, CD4 and CD8 IHC staining in lymphoid aggregate structures located within the stroma of anterior prostate tumour tissue from *Pten^Δ/Δ^* and *Pten^Δ/Δ^ BRF1^Tg^* mice (n=5 for each genotype). Scale bars are shown (100 µm for all images).

**Figure S6: Analysis of B cell markers revealed no significant difference in B cell infiltration within the stroma of *Pten^Δ/Δ^ BRF1^Tg^* compared to *Pten^Δ/Δ^* mice.**

Representative micrographs (left panels) of *Cd19*, *Cd79a*, *Pax5* RNA ISH and B220 IHC in prostate tumour tissue from *Pten^Δ/Δ^* and *Pten^Δ/Δ^ BRF1^Tg^* mice (n=5 for each genotype). Scale bars = 100 µm in all images. For all ISH and IHC markers, total observable prostate stromal tissue per slide was manually annotated on each sample from *Pten^Δ/Δ^* (n=5) and *Pten^Δ/Δ^* *BRF1^Tg^* (n=5) mice then staining within these annotated areas was analysed using HALO software (see methods). Individual data points are shown in the presented graphs (right panels); long horizontal lines indicate the Mean; error bars represent SEM.

**Figure S7: Analysis of immune aggregate structures revealed no significant difference in total immune aggregate area, plasma B cell, follicular dendritic cell, germinal centre or regulatory T cell markers in prostate tumours from *Pten^Δ/Δ^ BRF1^Tg^* compared to *Pten^Δ/Δ^* mice.**

(**A**) Representative micrographs of H+E staining (left panels) in anterior prostate tissue from end point tumour samples from *Pten^Δ/Δ^* and *Pten^Δ/Δ^ BRF1^Tg^* mice (n = 5 for each genotype). Scale bars are shown (1 mm). Immune aggregate structures (indicated by yellow arrows) and total prostate tissue on each slide from *Pten^Δ/Δ^* (n=5) and *Pten^Δ/Δ^* *BRF1^Tg^* (n=5) mice were manually annotated then their respective areas were calculated using HALO software. The ratio of these areas is presented in the graph shown (right panel). (**B**) Representative micrographs of CD138, CD21 and GL7 IHC staining (left panels) in immune aggregates located within the stroma of anterior prostate tissue from end point tumour samples from *Pten^Δ/Δ^* and *Pten^Δ/Δ^ BRF1^Tg^* mice (n = 5 for each genotype). Scale bars are shown (50 µm for CD138 IHC; 100 µm for CD21 and GL7 IHC). For all IHC markers, total observable immune aggregates per slide were manually annotated on each sample from *Pten^Δ/Δ^* (n=5) and *Pten^Δ/Δ^* *BRF1^Tg^* (n=5) mice then CD138, CD21 and GL7 staining within these annotated areas was analysed using HALO software (see methods) and is presented in the graphs shown (right panels). (**C**) Representative micrographs of FOXP3 IHC staining (left panels) in immune aggregates located within the stroma of anterior prostate tissue from end point tumour samples from *Pten^Δ/Δ^* and *Pten^Δ/Δ^ BRF1^Tg^* mice (n = 5 for each genotype). Scale bars are shown (100 µm). Total observable prostate stromal tissue per slide was manually annotated on each sample from *Pten^Δ/Δ^* (n=5) and *Pten^Δ/Δ^* *BRF1^Tg^* (n=5) mice then FOXP3 staining within these annotated areas was analysed using HALO software (see methods) and is presented in the graph shown (right panel). In (**A**-**C**), individual data points are shown in the presented graphs (right panels); long horizontal lines indicate the Mean; error bars represent SEM.

**Figure S8: Expression of *C7* inversely correlates with *BRF1* in TCGA (provisional) dataset and positively correlates with *CFD* and the T cell markers, *CD3E* and *CD4* in MSKCC (2010) and TCGA (provisional) datasets.**

(**A**) Comparison of incorporation of ^35^S-methionine label, which was measured by scintillation counting, between parental PC3 and Ctrl CL1, 2 cells (n=3; 2-3 wells per cell line were used in each individual experiment). Data from CL1 and CL2 cells was normalised to the mean of parental PC3 cells. Individual data points are shown in the presented graphs; long horizontal line indicates the Mean; error bars represent SEM. (**B**) Schematic outlining workflow for secretome study. PC3 Ctrl CL2 and BRF1 CL4, 5 and 6 were each labelled with both medium (MED.) and heavy arginine and lysine. Cells were serum starved for 72 hr prior then proteins were extracted from collected conditioned media. Samples were mixed in a 1:1 ratio as outlined to give both forward and reverse conditions, resolved by SDS-PAGE then analysed by mass spectrometry. (**C**) Oncoprint and heat map of *C7* expression in MSKCC (2010) prostate adenocarcinoma dataset. Patients were divided into low (n=68) and high (n=12) *C7* expressers based on z score < - 1.5 for survival analysis presented in Figure 4I. (**D**) Oncoprint and heat map of *C7* expression in TCGA (provisional) prostate adenocarcinoma dataset. Patients were divided into low (n=474) and high (n=18) *C7* expressers based on z score < -0.5 for survival analysis presented in Figure 4J. (**E-K**) Scatter plots showing the correlation of: *C7* with *BRF1* expression in TCGA (provisional) dataset (**E**); *C7* with *CFD* expression in MSKCC (2010) dataset (**F**); *C7* with *CFD* expression in TCGA (provisional) dataset (**G**); *C7* with *CD3E* expression in MSKCC (2010) dataset (**H**); *C7* with *CD3E* expression in TCGA (provisional) dataset (**I**); *C7* with *CD4* expression in MSKCC (2010) dataset (**J**) and *C7* with *CD4* in TCGA (provisional) dataset (**K**). For both datasets, all cases with mRNA expression data were evaluated [n=150 for MSKCC (2010); n=498 for TCGA (provisional)]. Pearson correlation coefficients and p values for all comparisons are stated in the main text.

**Supplementary Tables**

|  | **Number** | **Median**  **BRF1 Histoscore** | **Statistical Analysis** |
| --- | --- | --- | --- |
| **BPH** | 134 | 65 |  |
| **PCa** | 516 | 85.45 |  |
| **Mann Whitney t Test:**  **BPH vs PCa**  **(p value)** |  |  | 0.0032(**) |

**Table S1** – Number of cases for each cohort, median BRF1 histoscore and Mann Whitney t test results for comparison of the two cohorts (Figure 1B).

|  | **Number** | **Median**  **BRF1 Histoscore** | **Statistical Analysis** |
| --- | --- | --- | --- |
| **BPH** | 134 | 65 |  |
| **Gleason Sum Score < 7** | 120 | 80 |  |
| **Gleason Sum Score = 7** | 127 | 85.9 |  |
| **Gleason Sum Score > 7** | 187 | 88.3 |  |
| **Mann Whitney t Test:**  **BPH vs Gleason Sum Score < 7**  **(p value)** |  |  | ns |
| **Mann Whitney t Test:**  **BPH vs Gleason Sum Score = 7 (p value)** |  |  | 0.0039(**) |
| **Mann Whitney t Test:**  **BPH vs Gleason Sum Score > 7 (p value)** |  |  | 0.0091(**) |

**Table S2 –** Number of cases for each cohort, median BRF1 histoscore and Mann Whitney t test results for comparison of the two cohorts (Figure 1C). ns: not significant.

|  | **Number** | **Median Survival**  **(Years)** | **Statistical Analysis** |
| --- | --- | --- | --- |
| **Low BRF1** | 128 | 7.81 |  |
| **High BRF1** | 137 | 5.39 |  |
| **Log-rank (Mantel-Cox) Test:**  **Low BRF1 vs. High BRF1**  **(Chi square)** |  |  | 6.364 |
| **Log-rank (Mantel-Cox) Test:**  **Low BRF1 vs. High BRF1**  **(P value)** |  |  | 0.012 (*) |

**Table S3** – Number of cases, median overall survival (years) and Log-rank (Mantel-Cox) Test results (Chi square and p value) of survival curve comparison between patients segregated on the basis of having low (below median histoscore) or high (above median histoscore) BRF1 expression (Figure 1D).

|  | **Number** | **Median**  **Progression-Free Survival** | **Statistical Analysis** |
| --- | --- | --- | --- |
| **Low *BRF1*** | 68 | Undefined |  |
| **High *BRF1*** | 12 | 5.388 |  |
| **Log-rank (Mantel-Cox) Test:**  **Low vs High *BRF1***  **(Chi square)** |  |  | 5.37 |
| **Log-rank (Mantel-Cox) Test:**  **Low vs High *BRF1***  **(p value)** |  |  | 0.0205(*) |

**Table S4** – Number of cases, progression-free survival (years) and Log-rank (Mantel-Cox) Test results (Chi square and p value) of survival curve comparison between patients divided into low and high *BRF1* expressers based on z score > 2 in MSKCC (2010) dataset (Figure S1B).

|  | **Number** | **Median**  **Progression-Free Survival** | **Statistical Analysis** |
| --- | --- | --- | --- |
| **Low *BRF1*** | 474 | Undefined |  |
| **High *BRF1*** | 18 | 3.332 |  |
| **Log-rank (Mantel-Cox) Test:**  **Low vs High *BRF1***  **(Chi square)** |  |  | 4.367 |
| **Log-rank (Mantel-Cox) Test:**  **Low vs High *BRF1***  **(p value)** |  |  | 0.0366(*) |

**Table S5** – Number of cases, progression-free survival (years) and Log-rank (Mantel-Cox) Test results (Chi square and p value) of survival curve comparison between patients divided into low and high *BRF1* expressers based on z score > 2 in TCGA (Provisional) dataset (Figure S1D).

| **Cell Line** | **Comparison** | **p value**  **(Welch’s t test – unpaired, 2 tailed)** |
| --- | --- | --- |
| PC3 | eGFP-EV vs eGFP-BRF1 | <0.0001 |
| PC3 | HA-EV vs HA-BRF1 | <0.0001 |
| PC3M | eGFP-EV vs eGFP-BRF1 | <0.0001 |
| PC3M | HA-EV vs HA-BRF1 | 0.1144 |
| DU145 | eGFP-EV vs eGFP-BRF1 | 0.0214 |
| DU145 | HA-EV vs HA-BRF1 | 0.0202 |

**Table S6** – Statistical analysis of data relating to Figure S2A.

| **Cell Line** | **Comparison** | **p value**  **(Welch’s t test – unpaired, 2 tailed)** | **Adjusted p value (p.adj)**  **(Bonferroni correction)** |
| --- | --- | --- | --- |
| PC3 | NT siRNA2 vs BRF1 siRNA1 | 0.000043 | 0.000129 |
| PC3 | NT siRNA2 vs BRF1 siRNA2 | 0.000007 | 0.000021 |
| PC3 | NT siRNA2 vs BRF1 siRNA3 | 0.019775 | 0.059325 |
| PC3M | NT siRNA2 vs BRF1 siRNA1 | 0.247626 | 0.742878 |
| PC3M | NT siRNA2 vs BRF1 siRNA2 | 0.000012 | 0.000036 |
| PC3M | NT siRNA2 vs BRF1 siRNA3 | 0.00499 | 0.01497 |
| DU145 | NT siRNA2 vs BRF1 siRNA1 | <0.000001 | <0.000003 |
| DU145 | NT siRNA2 vs BRF1 siRNA2 | <0.000001 | <0.000003 |
| DU145 | NT siRNA2 vs BRF1 siRNA3 | 0.000013 | 0.000039 |
| LNCaP | NT siRNA2 vs BRF1 siRNA1 | 0.009045 | 0.027135 |
| LNCaP | NT siRNA2 vs BRF1 siRNA2 | 0.000002 | 0.000006 |
| LNCaP | NT siRNA2 vs BRF1 siRNA3 | <0.000001 | <0.000003 |
| LNCaP-AI | NT siRNA2 vs BRF1 siRNA1 | 0.002211 | 0.006633 |
| LNCaP-AI | NT siRNA2 vs BRF1 siRNA2 | <0.000001 | <0.000003 |
| LNCaP-AI | NT siRNA2 vs BRF1 siRNA3 | 0.000191 | 0.000573 |

**Table S7** – Statistical analysis of data relating to Figure S2B

|  | **Number** | **Median Survival**  **(Days)** | **Statistical Analysis** |
| --- | --- | --- | --- |
| ***Pten^Δ/Δ^*** | 12 | 316 |  |
| ***Pten^Δ/Δ^ BRF1^Tg^*** | 14 | 256 |  |
| **Log-rank (Mantel-Cox) Test:**  ***Pten^Δ/Δ^* vs *Pten^Δ/Δ^ BRF1^Tg^***  **(Chi square)** |  |  | 21.52 |
| **Log-rank (Mantel-Cox) Test:**  ***Pten^Δ/Δ^* vs *Pten^Δ/Δ^ BRF1^Tg^***  **(P value)** |  |  | < 0.0001 (****) |

**Table S8** – Number of mice for specified genotypes, median survival (days) and Log-rank (Mantel-Cox) Test results (Chi square and p value) of survival curve comparison between *Pten^Δ/Δ^* and *Pten^Δ/Δ^ BRF1^Tg^* mice (Figure 3C).

|  | **Number** | **Wet Weight (WW) (g)** | **Dry Weight (DW) (g)** |
| --- | --- | --- | --- |
| ***Pten^Δ/Δ^*** | 12 | 3.767 ± 0.5393 | 1.117 ± 0.1537 |
| ***Pten^Δ/Δ^ BRF1^Tg^*** | 14 | 3.757 ± 0.5579 | 1.064 ± 0.1324 |
| **Welch’s T Test:**  ***Pten^Δ/Δ^* vs *Pten^Δ/Δ^ BRF1^Tg^***  **(p values)** |  | 0.9903 | 0.7986 |

**Table S9** – Number of mice for specified genotypes, mean weights ± SEM and T Test results for end point prostate samples (Figure 3D).

**Table S10** – Shown in a separate Excel file are the 174 genes identified from analysis of RNA-Seq data as being significantly (fold change ≥ 1.5; p.adj ≤ 0.05) altered in *Pten^Δ/Δ^* *BRF1^Tg^* compared to *Pten^Δ/Δ^* tumours that are related to immune/inflammatory response. Highlighted in red in the table are complement factor 7 (*C7*) and *Cd4*.

| Processes | No. genes differentially expressed in dataset/Total no. genes known to be associated with these processes as characterised by Gene Ontology Consortium. | FDR |
| --- | --- | --- |
| cell adhesion | 115 / 1172 | 1.556E-43 |
| biological adhesion | 115 / 1195 | 5.639E-43 |
| inflammatory response | **85 / 811** | **2.327E-33** |
| response to external stimulus | 173 / 3214 | 2.686E-33 |
| immune system process | **190 / 3835** | **1.174E-32** |
| leukocyte chemotaxis | **46 / 202** | **5.820E-32** |
| extracellular matrix organization | 63 / 444 | 8.200E-32 |
| extracellular structure organization | 63 / 445 | 8.212E-32 |
| positive regulation of cell motility | 79 / 742 | 1.108E-31 |
| positive regulation of locomotion | 80 / 788 | 1.027E-30 |

**Table S11** – Top 10 processes identified from analysis of RNA-Seq data as being significantly (fold change ≥ 1.5; p.adj ≤ 0.05) altered in *Pten^Δ/Δ^* *BRF1^Tg^* compared to *Pten^Δ/Δ^* tumours. FDR = false discovery rate. Immune-related processes are highlighted red in the table.

**Table S12** – Shown in a separate Excel file are the 52 genes identified from analysis of proteomics data as being significantly (fold change ≥ 1.5; p.adj ≤ 0.05) altered in *Pten^Δ/Δ^* *BRF1^Tg^* compared to *Pten^Δ/Δ^* tumours that are related to the immune process GO term.

| Processes | No. proteins differentially changed in dataset/Total no. proteins known to be associated with these processes as characterised by Gene Ontology Consortium. | FDR |
| --- | --- | --- |
| catabolic process | 90 / 2642 | 2.212E-14 |
| leukocyte degranulation | **41 / 657** | **2.664E-13** |
| small molecule metabolic process | **82 / 2434** | **6.245E-13** |
| myeloid leukocyte mediated immunity | **41 / 687** | **6.245E-13** |
| myeloid leukocyte activation | **43 / 773** | **8.571E-13** |
| neutrophil activation | **39 / 636** | **8.571E-13** |
| cellular catabolic process | 78 / 2282 | 8.571E-13 |
| granulocyte activation | **39 / 640** | **8.571E-13** |
| neutrophil mediated immunity | **39 / 642** | **8.571E-13** |
| myeloid cell activation involved in immune response | **40 / 679** | **9.075E-13** |

**Table S13** – Top 10 processes identified from analysis of proteomics data as being significantly (fold change ≥ 1.5; p.adj ≤ 0.05) altered in *Pten^Δ/Δ^* *BRF1^Tg^* compared to *Pten^Δ/Δ^* tumours. FDR = false discovery rate. Immune-related processes are highlighted red in the table.

**Table S14** – Shown in a separate Excel file are the top 10 pathways significantly (p.adj ≤ 0.05 in compared data) altered at both RNA-Seq and proteomics level. Highlighted in red is the complement cascade.


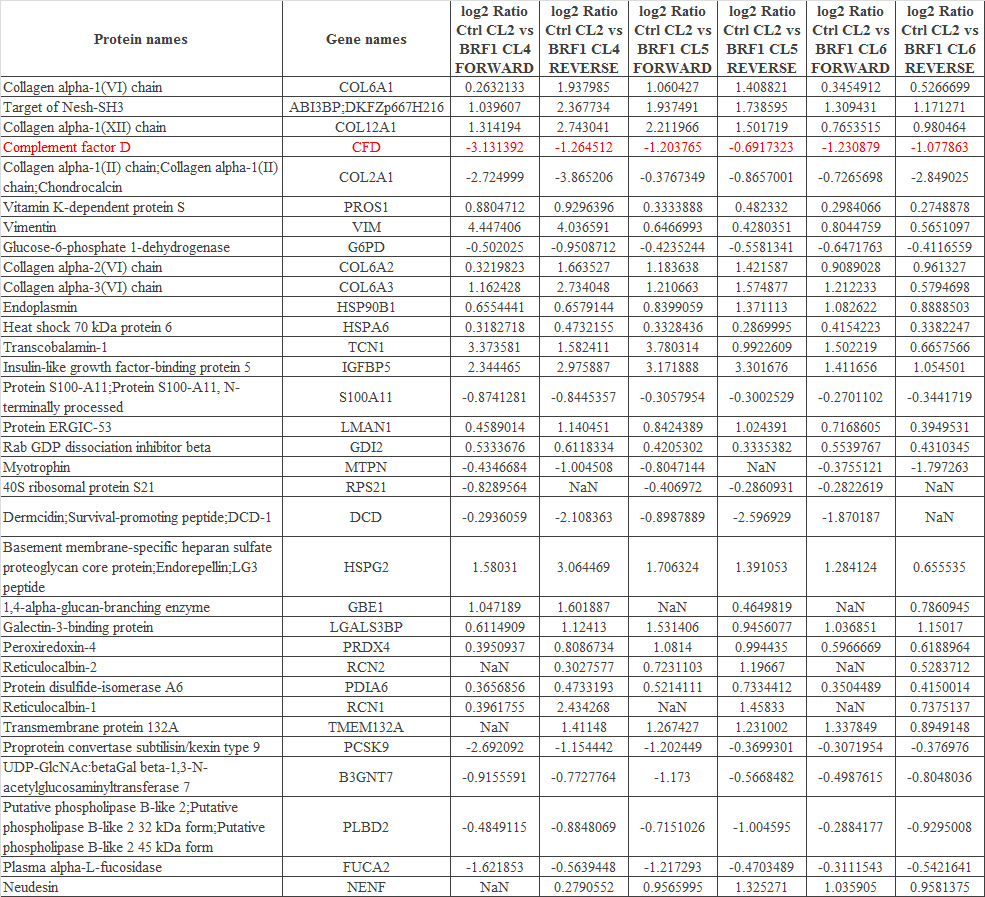


**Table S15 –** Proteins whose abundance was ≥ 1.2 fold changed in the same direction in PC3 BRF1 CL4, CL5 and CL6 when compared to Ctrl CL2. NaN indicates no SILAC ratio could be calculated. Highlighted in red is complement factor D (CFD).

|  | **Number** | **Median**  **Progression-Free Survival** | **Statistical Analysis** |
| --- | --- | --- | --- |
| **High *C7*** | 66 | Undefined |  |
| **Low *C7*** | 14 | 5.388 |  |
| **Log-rank (Mantel-Cox) Test:**  **BPH vs PCa**  **(Chi square)** |  |  | 6.185 |
| **Log-rank (Mantel-Cox) Test:**  **BPH vs PCa**  **(p value)** |  |  | 0.0129(*) |

**Table S16** – Number of cases, median overall survival (years) and Log-rank (Mantel-Cox) Test results (Chi square and p value) of survival curve comparison between patients divided into low and high *C7* expressers based on z score < -1.5 in MSKCC (2010) dataset (Figure 7D).

|  | **Number** | **Median**  **Progression-Free Survival** | **Statistical Analysis** |
| --- | --- | --- | --- |
| **High *C7*** | 474 | Undefined |  |
| **Low *C7*** | 18 | 6.113 |  |
| **Log-rank (Mantel-Cox) Test:**  **BPH vs PCa**  **(Chi square)** |  |  | 12.25 |
| **Log-rank (Mantel-Cox) Test:**  **BPH vs PCa**  **(p value)** |  |  | 0.0005(***) |

**Table S17** – Number of cases, median overall survival (years) and Log-rank (Mantel-Cox) Test results (Chi square and p value) of survival curve comparison between patients divided into low and high *C7* expressers based on z score < -0.5 in TCGA (Provisional) dataset (Figure 7F).

| **Oligo siRNApair name** | **5’ to 3’ sequence** |
| --- | --- |
| **BRF1 siRNA1**  (Thermo Fisher Scientific, Silencer® Select, s6323) | Sense GGCUCACGGAAUUUGAAGATT  Anti UCUUCAAAUUCCGUGAGCCTC |
| **BRF1 siRNA2**  (Thermo Fisher Scientific, Silencer® Select, s223824) | Sense GCCAGAAUGCAUGACUUCATT  Anti UGAAGUCAUGCAUUCUGGCTG |
| **BRF1 siRNA3**  (Thermo Fisher Scientific, Silencer® Select, s194479) | Sense CACCAGUCAGUUGACCAUUTT  Anti AAUGGUCAACUGACUGGUGGG |
| **NTsiRNA2 (Non Targeting)**  (GE Healthcare, Dharmacon, D-001810-10) | Not disclosed |
| **NTsiRNA3 (All Stars)**  (Qiagen, SI03650318) | Not disclosed |

**Table S18 –** List of siRNA Sequences

| **Gene** | **Forward Primer** | **Reverse Primer** | **UPL Probe** |
| --- | --- | --- | --- |
| **h*BRF1*** | 5’-gcaagagagctctgcatcaat-3’ | 5’-atggacacctcgtggttctt-3’ | 62 |
| ***Casc3*** | 5’-cacctcctcatctgtatcctaaca-3’ | 5’-ctgggcggggttatagtaagt-3’ | 25 |
| ***C7*** | 5’-tgccaagtgtgtgcaaaaag-3’ | 5’-agtttctcccaaggctgaca-3’ | 22 |

**Table S19 –** Primer pair sequences and UPL probe used for each gene studied.

| **Clinico-pathological characteristics** | **Patient Numbers n (%)**  **Total n = 516** |
| --- | --- |
| **Age at Diagnosis**  (Cases with data / Missing) | 414 (80.2%) / 102 (19.8%) |
| **Gleason Grade**  (< 7 / 7 / >7 / Missing) | 120 (23.3%) / 127 (24.6%) / 187 (36.2%) / 82 (15.9%) |
| **T Stage**  (T1-2 / T3-T4 / Missing) | 214 (41.5%) / 135 (26.2%) / 167 (32.4%) |
| **Serum PSA Concentration (ng/mL) at diagnosis**  (< 10 / 10-20 / > 20 / Missing) | 73 (14.1%) / 45 (8.7%) / 90 (17.4%) / 308 (59.7%) |
| **Metastases**  (No / Yes / Missing) | 72 (14%) / 45 (8.7%) / 399 (77.3%) |

**Table S20 –** Summary of data available to evaluate clinico-pathological characteristics of PCa TMA cohort.

**Supplementary References**

1 Loveridge CJ, Mui EJ, Patel R, Tan EH, Ahmad I, Welsh M *et al*. Increased T-cell Infiltration Elicited by Erk5 Deletion in a Pten-Deficient Mouse Model of Prostate Carcinogenesis. *Cancer Res* 2017; 77: 3158-3168.

2 Ahmad I, Mui E, Galbraith L, Patel R, Tan EH, Salji M *et al*. Sleeping Beauty screen reveals Pparg activation in metastatic prostate cancer. *Proc Natl Acad Sci U S A* 2016; 113: 8290-8295.

3 Cerami E, Gao J, Dogrusoz U, Gross BE, Sumer SO, Aksoy BA *et al*. The cBio cancer genomics portal: an open platform for exploring multidimensional cancer genomics data. *Cancer Discov* 2012; 2: 401-404.

4 Gao J, Aksoy BA, Dogrusoz U, Dresdner G, Gross B, Sumer SO *et al*. Integrative analysis of complex cancer genomics and clinical profiles using the cBioPortal. *Sci Signal* 2013; 6: pl1.

5 Taylor BS, Schultz N, Hieronymus H, Gopalan A, Xiao Y, Carver BS *et al*. Integrative genomic profiling of human prostate cancer. *Cancer Cell* 2010; 18: 11-22.

6 Halkidou K, Gnanapragasam VJ, Mehta PB, Logan IR, Brady ME, Cook S *et al*. Expression of Tip60, an androgen receptor coactivator, and its role in prostate cancer development. *Oncogene* 2003; 22: 2466-2477.

7 Graczyk D, White RJ, Ryan KM. Involvement of RNA Polymerase III in Immune Responses. *Mol Cell Biol* 2015; 35: 1848-1859.

8 Sutcliffe JE, Brown TR, Allison SJ, Scott PH, White RJ. Retinoblastoma protein disrupts interactions required for RNA polymerase III transcription. *Mol Cell Biol* 2000; 20: 9192-9202.

9 Johnston IM, Allison SJ, Morton JP, Schramm L, Scott PH, White RJ. CK2 forms a stable complex with TFIIIB and activates RNA polymerase III transcription in human cells. *Mol Cell Biol* 2002; 22: 3757-3768.

10 Bronson SK, Plaehn EG, Kluckman KD, Hagaman JR, Maeda N, Smithies O. Single-copy transgenic mice with chosen-site integration. *Proc Natl Acad Sci U S A* 1996; 93: 9067-9072.

11 Tucker KL, Wang Y, Dausman J, Jaenisch R. A transgenic mouse strain expressing four drug-selectable marker genes. *Nucleic Acids Res* 1997; 25: 3745-3746.

12 Nagy A. *Manipulating the mouse embryo : a laboratory manual*. Cold Spring Harbor Laboratory Press: Cold Spring Harbor, N.Y., 2003.

13 Church DM, Schneider VA, Graves T, Auger K, Cunningham F, Bouk N *et al*. Modernizing reference genome assemblies. *PLoS Biol* 2011; 9: e1001091.

14 Kim D, Pertea G, Trapnell C, Pimentel H, Kelley R, Salzberg SL. TopHat2: accurate alignment of transcriptomes in the presence of insertions, deletions and gene fusions. *Genome Biol* 2013; 14: R36.

15 Langmead B, Salzberg SL. Fast gapped-read alignment with Bowtie 2. *Nat Methods* 2012; 9: 357-359.

16 Anders S, Huber W. Differential expression analysis for sequence count data. *Genome Biol* 2010; 11: R106.

17 McGarry DJ, Shchepinova MM, Lilla S, Hartley RC, Olson MF. A Cell-Permeable Biscyclooctyne As a Novel Probe for the Identification of Protein Sulfenic Acids. *ACS Chem Biol* 2016; 11: 3300-3304.

18 Rappsilber J, Mann M, Ishihama Y. Protocol for micro-purification, enrichment, pre-fractionation and storage of peptides for proteomics using StageTips. *Nat Protoc* 2007; 2: 1896-1906.

19 Knight JR, Bastide A, Peretti D, Roobol A, Roobol J, Mallucci GR *et al*. Cooling-induced SUMOylation of EXOSC10 down-regulates ribosome biogenesis. *RNA* 2016; 22: 623-635.
